# High-quality chromosome-level genome assembly of female *Artemia franciscana* reveals sex chromosome and *Hox* gene organization

**DOI:** 10.1016/j.heliyon.2024.e38687

**Published:** 2024-09-28

**Authors:** Euna Jo, Minjoo Cho, Soyun Choi, Seung Jae Lee, Eunkyung Choi, Jinmu Kim, Jang Yeon Kim, Sooyeon Kwon, Jun Hyuck Lee, Hyun Park

**Affiliations:** a Division of Biotechnology, College of Life Sciences and Biotechnology, Korea University, Seoul, 02841, South Korea; b Division of Life Sciences, Korea Polar Research Institute, Incheon, 21990, South Korea

**Keywords:** Brine shrimp, Long-read sequencing, Hi-C scaffolding, Gene annotation, ZW chromosome, Sex determination, Homeobox gene

## Abstract

*Artemia* is a crustacean genus belonging to the order Anostraca in the class Branchiopoda and lives in inland hypersaline lakes. Among the genus, *A. franciscana* is a valuable species as a fish food in the aquaculture industry or as an aquatic model organism for toxicity tests. However, genomic data for *A. franciscana* remains incomplete. In this study, high-quality genome assembly at the chromosome level of female *A. franciscana* was conducted by combining various sequencing and assembly technologies. The final *A. franciscana* assembled genome was 1.27 Gb in length, containing 21 chromosomal scaffolds (>10 Mb). The scaffold N50 was 45.3 Mb, with a complete BUSCO value of 91.0 %, thereby confirming that a high-quality genome was assembled. Gene annotation shows that the *A. franciscana* genome contained 67.26 % of repetitive sequences, and a total of 26,923 protein-coding genes were predicted. Among the 21 chromosome-scale scaffolds, chromosome 1 was identified as a sex chromosome Z. Additionally, five contigs of putative W chromosome fragments and the candidate sex-determining genes were suggested. Ten homeobox (*Hox*) genes were identified in *A. franciscana* on the chromosome 14, which were in two subclusters with a large gap. *Hox* gene organizations within 13 arthropods showed that four anostracans had conserved synteny. This study provides a new female *Artemia* genome with sex chromosome and the first complete genomic arrangement of the *Hox* cluster in Anostraca. This study will be a useful genomic and genetic reference for understanding the evolution and development of *A. franciscana*.

## 1 Introduction

The brine shrimp *Artemia* is an aquatic crustacean that is widely distributed worldwide, except in Antarctica [[1], [2], [3], [4]]. Currently, nine species have been reported in the genus *Artemia*, including the newly reported species [5]. Three species of the genus, *A. franciscana*, *A. monica*, and *A. persimilis*, are native to the American Continent (New World), while Eurasia, Africa, and Australia (Old World) contain the other six sexual species alongside many obligate parthenogenetic lineages [2,[5], [6], [7], [8], [9], [10]]. Halophilic *Artemia* mainly inhabits coastal or inland salt lakes and is a keystone species in the food web of hypersaline ecosystems, where prokaryotes are relatively abundant and eukaryotes are rare [11,12]. *Artemia* has been regarded as a non-selective filter feeder that consumes a broad spectrum of food—for example: protozoa, microalgae, and bacteria [13,14]. Other characteristic of *Artemia* is their unique life cycle, which has two reproductive modes, oviparous (diapaused cysts) and ovoviviparous (free-swimming nauplii) that can be influenced by environmental factors, such as photoperiod, salinity, temperature, and oxygen levels [[15], [16], [17]].

Species of *Artemia*, especially *A. franciscana*, have been important in the aquaculture industry because they are easily hatched from dormant cysts and the nauplii can be used as part of a nutritious diet for fish [13,[18], [19], [20]]. Moreover, they are widely used for ecotoxicity tests as aquatic model organisms [[21], [22], [23], [24]]. However, despite these interesting traits and the significance of *Artemia*, genomic resources have remained incomplete until recently. Through the years, several attempts have been made to uncover the *A. franciscana* genome using amplified fragment length polymorphism (AFLP) and specific-locus amplified fragment (SLAF)-based genetic linkage maps [25,26]. Presently, genome assembly of *A. franciscana* has been reported and addressed its features as an extremophile [16]. However, the genome remains fragmented, suggesting a more complete and contiguous genome assembly is needed to reveal other important structural and functional properties. The first chromosome-level genome assembly in *Artemia* species, *A. sinica* genome, has been recently published, followed by *A. franciscana* genome, providing detailed characterized sex chromosomal evolution across this genus [27,28]. Nevertheless, they have limitations, whereby they did not present any evolutionary nor functional aspect of annotated genes.

The genus *Artemia* has female heterogametic sex chromosomes, meaning females have ZW chromosomes and males have ZZ chromosomes, with a ZW sex-determination system [25,29,30]. Primary sex determination genes that are located on sex chromosomes lead to the development of either female or male gonads [31]. In addition to the study by Elkrewi et al. [27], there have been several studies attempting to identify sex-determining regions or genes in *Artemia* [25,30,32,33], although the primary sex-determining genes involved and their location are still unknown. The master sex-determining genes in *Artemia* are thought to be different from those in other animals, yet further research is required to confirm this hypothesis. Representative primary sex-determining genes in animals, such as the sex-determining region of the Y chromosome (*SRY*) gene in mammals [34] and the Z-linked doublesex and mab-3-related transcription factor 1 (*Dmrt1*) gene in birds [35], are usually located on one of the sex chromosomes. Therefore, obtaining a high-quality female *Artemia* genome is of the utmost importance in order to investigate both Z and W sex chromosomes and determine the sex-determination region.

The homeobox (*Hox*) genes encode homeodomain transcription factors. These genes play an important role in embryonic development by determining the identities of the segments along the anterior–posterior (A–P) axis of the embryo [36]. Since *Hox* genes were first discovered in *Drosophila*, they have subsequently been found in various eukaryotes, including animals, fungi, plants, and protists [[37], [38], [39], [40], [41]]. In arthropods, ten canonical *Hox* genes are known to be relatively well-conserved and are typically found on a chromosome with a single complex [36,42]. These include labial (*lab*), proboscipedia (*pb*), *Hox3*, Deformed (*Dfd*), Sex combs reduced (*Scr*), fushi tarazu (*ftz*), Antennapedia (*Antp*), Ultrabithorax (*Ubx*), abdominal-A (*abd-A*), and Abdominal-B (*Abd-B*). Many of the previous studies conducted on *Hox* genes in *Artemia* have focused on the genes involved in the trunk segments [[43], [44], [45], [46], [47], [48], [49], [50]]. Thus, the presence of several anterior *Hox* genes (*lab*, *pb*, or *Hox3*) was marked as missing [[51], [52], [53]], until recent study showed that *A. franciscana* also possessed all ten canonical *Hox* genes [54]. However, the entire arrangement of the *Hox* cluster in *A. franciscana* was not identified due to limitations in the quality of genome assembly.

Here, new high-quality chromosome-level genome assembly and annotation of female *A. franciscana* were conducted by combining a variety of sequencing technologies and analytical tools. This study identified genomic characteristics and sex chromosomes, thereby suggesting the candidate sex-determining regions. Furthermore, the complete *Hox* gene cluster in *A. franciscana* was first identified with the comparison of genomic arrangements of *Hox* genes among arthropods.

## 2 Results

### 2.1 Genome sequencing and chromosome-level assembly

**Fig. 1:**
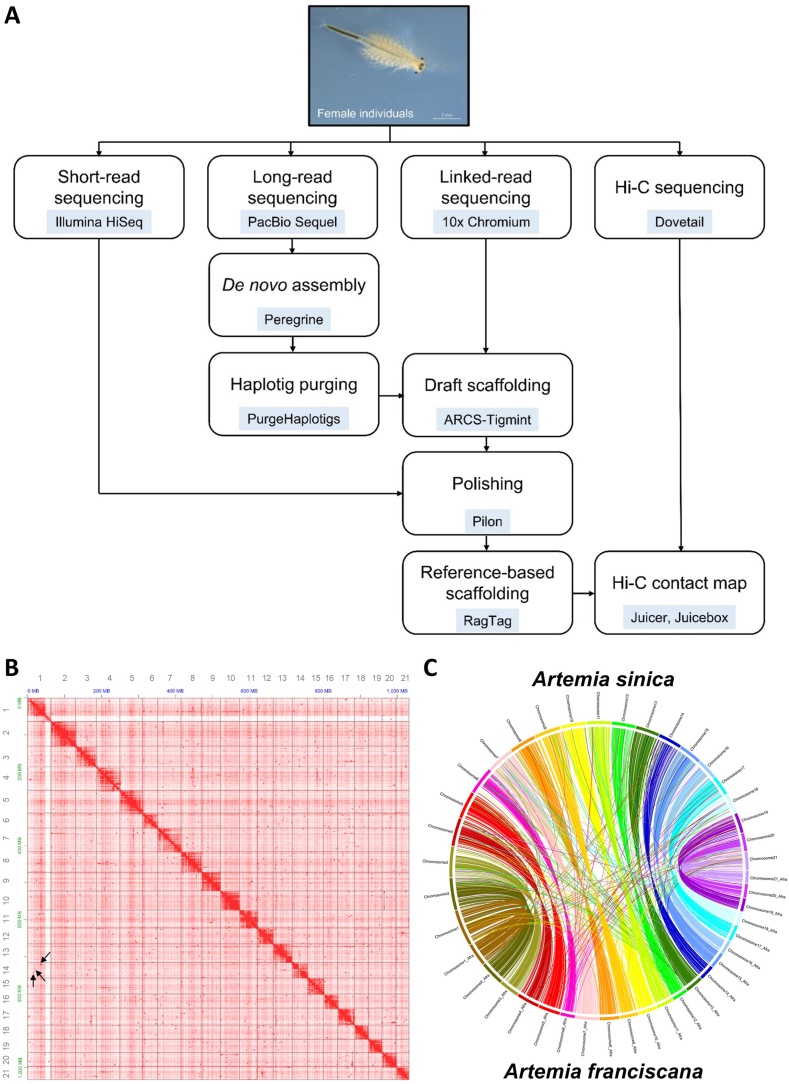
Chromosome-level genome assembly of *Artemia franciscana.***(A)** Workflow of the bioinformatic steps involved in the genome assembly of *A. franciscana*. **(B)** Hi-C contact map of 21 chromosome-length scaffolds in the *A. franciscana* genome. Darker red indicates higher chromatin interaction frequencies. Arrows represent examples of interchromosomal interaction signals. **(C)** Circos plot between *A. franciscana* and *A. sinica* genome assemblies. Connections within the circle represent syntenic relationships between the two assemblies. (For interpretation of the references to color in this figure legend, the reader is referred to the Web version of this article.)

The *A. franciscana* genome was sequenced using Illumina paired-end, PacBio single-molecule real-time (SMRT), 10× chromium linked-reads, and Dovetail high-throughput chromosome conformation capture (Hi-C) sequencing technologies (Fig. 1A). In addition to previously produced Illumina short-reads for genome survey [55], PacBio long-read sequencing was performed by Sequel platform using two SMRT cells and yielded 6,065,749 reads, with a total length of 599.09 Gb (Table S1). Moreover, 414 million 10× linked-reads and 686 million Dovetail Hi-C reads were generated by an Illumina Novaseq 6000 instrument, with total lengths of 62.55 Gb and 103.60 Gb, respectively (Table S1). The sequencing reads produced by these different methods were assembled using various assembly tools, and the summary statistics of intermediate genome assemblies are presented in Table S2.

After RagTag scaffolding with congeneric *A. sinica* genome assembly [27], the sex chromosomes were separated based on the female and male whole-genome sequencing (WGS) mapping results (further details are provided in ‘Identification of sex chromosome’ section). The final assembly of the *A. franciscana* genome was approximately 1.27 Gb (Table 1). The longest scaffold was ∼67.3 Mb, and the N50 length was 45.3 Mb (Table 1). The number of scaffolds in the assembled genome was 6,486, while 21 scaffolds found to be above 10 Mb in length were considered chromosomes (Fig. 1B; Table 1; Table S3). The 21 chromosome-level scaffolds had a total length of ∼1.03 Gb (Table S3) and were numbered in accordance with the *A. sinica* chromosomes. In the Hi-C heatmap, some interactions were observed between chromosomes, *e.g.*, chromosome 14 with chromosomes 1 (arrows in Fig. 1B). The syntenic relationships between the *A. franciscana* and *A. sinica* chromosomes showed a high level of synteny conservation through mostly one-to-one matching (Fig. 1C). Thereafter, the genome assembly statistics in this study were compared to the recently published *A. franciscana* genomes, GCA_019857095 [16] and GCA_036172535 [28] (Table 1). Benchmarking Universal Single-Copy Orthologs (BUSCO) v5.6.1 was used alongside the Eukaryota_odb10 database to assess the completeness of the *A. franciscana* genome assembly. Among 255 eukaryote gene sets, 232 (91.0 %) genes were completely identified, while 14 (5.5 %) genes were partially identified (Table S4). Among the complete eukaryotic genes, 217 (85.1 %) and 15 (5.9 %) were identified as single-copy and duplicated BUSCOs, respectively (Table S4). Quality value (QV) and k-mer completeness evaluated by Merqury, showed a QV of 47.62 and completeness of 68.81 % (Table S5, Fig. S1).

**Table 1:** Comparison of *Artemia franciscana* genome assembly statistics.

Assembly	This study	GCA_019857095 (De Vos et al., 2021)	GCA_036172535 (Bett et al., 2024)
Number of scaffolds	6486	20,887	2059
Total size of scaffolds (bp)	1,270,721,445	850,560,342	1,133,270,645
Longest scaffold (bp)	67,337,973	858,101	66,480,397
Number of scaffolds >10 Mb	21	–	21
Percentage of scaffolds in chromosomes	81 %	–	81 %
N50 scaffold length (bp)	45,252,357	112,377	43,077,890
L50 scaffold count	12	2122	11
N50 contig length (bp)	383,004	61,161	459,800
L50 contig count	919	3977	741
Scaffold GC content (%)	34.71	34.68	34.90

### 2.2 Repeat analysis and gene annotation

The repeat contents identified in the *A. franciscana* genome were analyzed, including the tandem repeats and transposable elements (TEs). A total of 67.26 % of the *A. franciscana* genome contained repeated sequences, with TEs accounting for 64.73 % (Table S6). Long interspersed elements (LINEs) were the most abundant TE, accounting for 12.75 % of the total, followed by rolling-circle transposons (Helitrons; 8.81 %), and DNA elements (7.16 %) (Table S6). The Kimura substitution level for each TE copy was calculated to estimate the age of the TEs. The results indicated that copies that recently diverged from the consensus sequence (Kimura distance K-values ≤3) were strongly shaped in the repeat landscape (Fig. S2).

A total of 26,923 protein-coding genes were predicted in the *A. franciscana* genome using the EVidenceModeler (EVM) pipeline by combining transcript mapping, *ab initio* gene predictions, and protein homology searches. The total length of the exons was ∼51.9 Mb and each gene contained an average of 5.65 exons (Table 2). BUSCO evaluation in transcriptome mode showed 82.0 % complete and 7.5 % fragmented BUSCOs, whereas 10.5 % were missing (Table S4). Functional annotation was conducted based on nr, Gene Ontology (GO), Kyoto Encyclopedia of Genes and Genomes (KEGG), SwissProt, Pfam, SignalP, TmHMM, and InterProScan databases. Among the predicted genes, a total of 26,821 (99.6 %) genes were functionally annotated in at least one database (Table 2). Among these, 24,319 (90.3 %) genes were annotated in the GO database (Table 2). The distributions of GO terms for each category of biological process (BP), molecular function (MF), and cellular component (CC) are shown in Fig. S3.

**Table 2:** Statistics of *Artemia franciscana* genome annotation.

Annotation database	Annotated number	Percentage (%)
No. of genes	26,923	
nr annotation	26,022	96.7
GO annotation	24,319	90.3
KEGG annotation	9988	37.1
Swissprot blastx annotation	18,808	69.9
Swissprot blastp annotation	18,685	69.4
Pfam annotation	17,827	66.2
SignalP annotation	25,251	93.8
TmHMM annotation	5480	20.4
InterProScan annotation	23,940	88.9
	**Count**	**Total length (bp)**
Exon	152,030	51,865,828
CDS	142,540	34,995,148

### 2.3 Gene family evolution

**Fig. 2:**
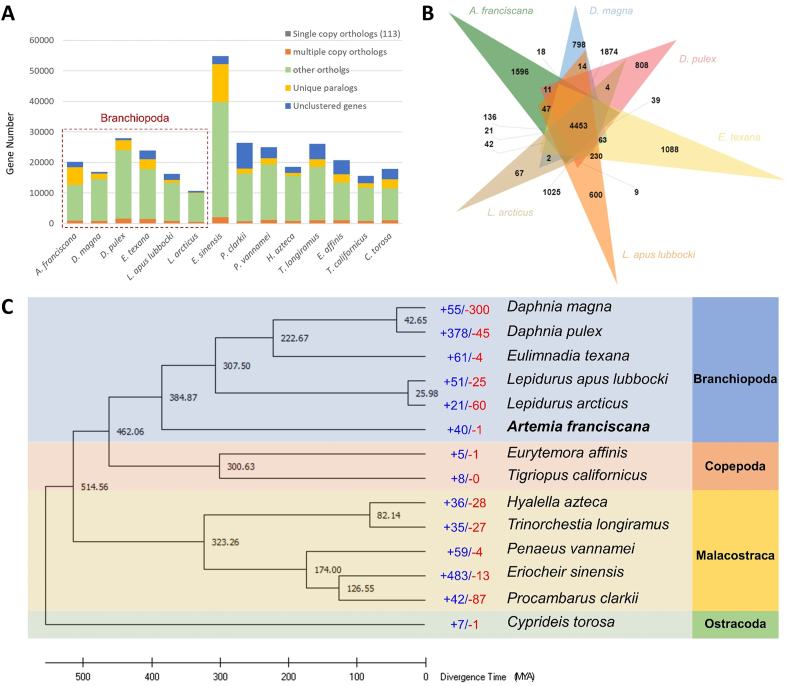
Gene family comparisons. **(A)** Orthologous gene families between *Artemia franciscana* and other crustacean species. **(B)** Venn diagram of orthologous gene families between *A. franciscana* and five branchiopod species. **(C)** Phylogeny and gene family gain-and-loss analyses of *Artemia franciscana* within the crustacean species. Numbers at each node indicate estimated divergence times between lineages. The number of significantly expanded (+) and contracted (−) gene families are shown in blue and red, respectively. (For interpretation of the references to color in this figure legend, the reader is referred to the Web version of this article.)

A total of 14 species, which were assigned to various taxa of Crustacea, were selected to analyze the gene family evolution (Table S7). In total, 26,152 orthologue gene families were identified, of which 2720 were shared in all examined species, with 113 single-copy orthologous genes (SCOGs) (Fig. 2A). A total of 908 species-specific orthogroups were found for *A. franciscana* across 14 species, which contained 5840 genes (Fig. 2A; Table S8). For the results of the OrthoVenn3 analysis for six species in the class Branchiopoda (*i.e.*, *A. franciscana*, *D. magna*, *D. pulex*, *Eulimnadia texana*, *Lepidurus apus lubbocki*, and *L. arcticus*), 4453 common orthologous gene families were identified, while 1596 were specific to the *A. franciscana* genome (Fig. 2B).

A phylogenetic tree was constructed for the 14 crustacean species using the 113 SCOGs and showed that the six species belonging to the class Branchiopoda form a monophyletic cluster (Fig. 2C). Among branchiopods, *A. franciscana* diverged from the other crustaceans the earliest, with the divergence time between *A. franciscana* and the other branchiopods calibrated in TimeTree being approximately 384.87 million years ago (Fig. 2C). Gene family gain-and-loss analysis using CAFE exhibited that the *A. franciscana* genome contained 40 significantly expanded gene families and one contracted gene family (Fig. 2C).

The GO enrichment analysis for the genes included in the *A. franciscana*-specifically expanded gene families revealed that they were involved in a broad range of gene functions, especially in the GO terms related to muscle cytoskeleton (larval somatic muscle development, GO:0007526; cortical actin cytoskeleton organization, GO:0030866; postsynaptic actin cytoskeleton organization, GO:0098974; structural constituent of postsynaptic actin cytoskeleton, GO:0098973; actin filament, GO:0005884) (Table S9).

### 2.4 Identification of sex chromosome

The SLAF markers from Han et al. [26] were aligned into the final genome assembly for *A. franciscana* to confirm the continuity between the linkage groups (LGs) and the chromosomes. The markers on 21 LGs were generally well-matched with the 21 chromosomes in the final assembly (Fig. S4). Most of the markers located on LG6, defined as a sex chromosome, were matched with chromosome 1 (Fig. S4). Chromosome 1 was also concluded as the Z sex chromosome in the *A. sinica* genome assembly [27], which was used as a reference for the RagTag scaffolding. Overall, the final assembly of the *A. franciscana* genome indicated that chromosome 1 is a sex chromosome.

**Fig. 3:**
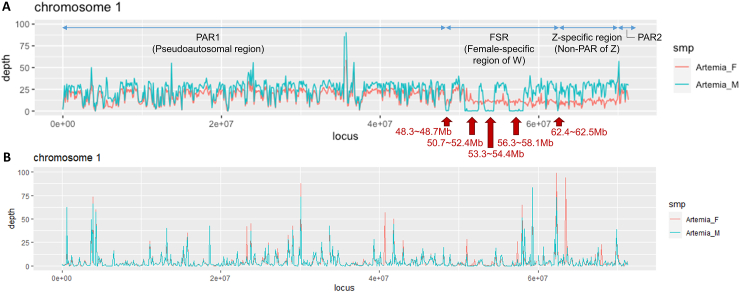
Comparison of coverage depths for chromosome 1. Patterns of whole genome sequencing **(A)** and whole transcriptome sequencing **(B)** for female and male *Artemia franciscana*. Scarlet color plots represent female mapping reads, and mint color plots represent male mapping reads. Red arrows indicate the female-specific regions in the W chromosome. (For interpretation of the references to color in this figure legend, the reader is referred to the Web version of this article.)

Subsequently, to determine the sex chromosome and compare the coverage patterns, both female and male whole-genome sequencing (WGS) reads were mapped to each chromosome using the Burrows–Wheeler Aligner (BWA). As a result, pseudoautosomal regions (PARs), female-specific regions (FSRs), and Z-specific regions were identified in chromosome 1, while there were no significant differences in the coverage between the female and male reads in the other chromosomes (Fig. 3A; Fig. S5). The PARs showed similar genomic coverage depths in the female (ZW) and male (ZZ) chromosomes, and the regions occupied up to ∼48.3 Mb (PAR1) and after 70 Mb (PAR2) of chromosome 1 (Fig. 3A). The FSRs were located from 48.3 to 62.5 Mb, and the regions where coverage was observed only in females (ZW), with no coverage in males (ZZ), were regarded as W chromosome fragments; they were found across the five segments (red arrows in Fig. 3A). To isolate ZW-mixed sex chromosome, the RagTag scaffold for chromosome 1 was re-examined based on the WGS mapping coverage. We identified five W contigs matching the position of FSRs (marked on Table S10), which were removed from chromosome 1 and left as separate W contigs. The regions between 62.5 Mb and 70 Mb, where the coverage depth in females was about half that for males, were considered as non-pseudoautosomal regions in the Z chromosome (Fig. 3A). In addition, whole transcriptome sequencing (WTS, RNA-Seq) reads for female and male *A. franciscana* were mapped by Bowtie2 to the 21 chromosomes in the reference genome to examine and compare the coverage patterns. The overall mapping coverage depths were similar between the female and male RNA-Seq reads, although differences were found in some regions (Fig. 3B; Fig. S6).

### 2.5 Genes in the female-specific regions and candidate sex-determining genes

**Fig. 4:**
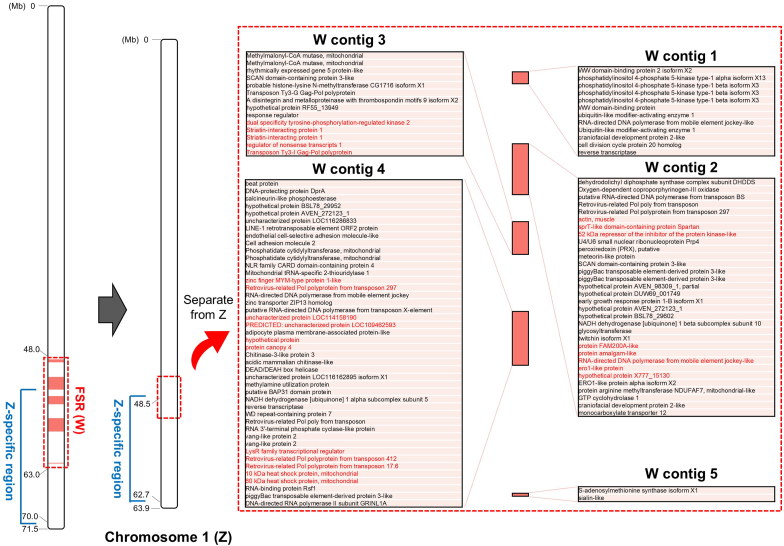
Female-specific regions (FSRs) of the W and Z-specific region in chromosome 1 of *Artemia franciscana*. Red colored genes in W contigs are those contained in regions with more than two-fold higher expression levels in females. (For interpretation of the references to color in this figure legend, the reader is referred to the Web version of this article.)

Putative W chromosome genes in *A. franciscana* were identified in the five W contigs using the annotation data (Fig. 4). Among these genes, several were positioned in the regions that female RNA-Seq reads mapped more than twice as many as in males, such as ‘transposon Ty3-I Gag-Pol polyprotein’, ‘transposons ‘RNA-directed DNA polymerase from mobile element jockey-like’, ‘10 kDa heat shock protein, mitochondrial’, and ‘60 kDa heat shock protein, mitochondrial’ (red letters in Fig. 4; Table S11). Some of these genes have been known to be associated with female-specific functions or sex chromosomes, which have been described in more detail in the Discussion section.

For the 23 genes presumed to be on the W chromosome of *A. franciscana*, we designed W chromosome-specific markers to confirm whether they are located on the W chromosome and distinguish females and males by PCR amplification (Table S12). PCR validation results showed that nine of the 23 marker sets yielded clear single PCR products in female DNA, while no PCR products were presented in male DNA (Fig. S7). The genes containing these markers, for which amplification was confirmed only in females, can be located on the W chromosome and have potential as sex-specific markers. The descriptions of the genes included the following: ‘actin, muscle’, ‘sprT-like domain-containing protein Spartan’, ‘52 kDa repressor of the inhibitor of the protein kinase-like’, ‘protein amalgam-like’, ‘ero1-like protein’, ‘Striatin-interacting protein 1’ (two copies), ‘Retrovirus-related Pol polyprotein from transposon 17.6’, and ‘60 kDa heat shock protein, mitochondrial’.

In addition to the W chromosome genes, the gene annotations were investigated in regions where the differences in the mapping coverage depths between female and male RNA-Seq data were more than doubled throughout the post-PAR1 (after 48.5 Mb). Among the genes located on the non-pseudoautosomal Z-specific region, seven genes had high coverage depths in males: ‘Methylmalonyl-CoA mutase, mitochondrial’, ‘rhythmically expressed gene 5 protein’, ‘Phosphatidate cytidylyltransferase, mitochondrial’, ‘Nucleotide-binding oligomerization domain-containing 1-like protein’, ‘Mitochondrial tRNA-specific 2-thiouridylase 1’, and two copies of the ‘chaoptin’ gene (Table S11).

### 2.6 Homeobox gene cluster in *Artemia franciscana*

In the *A. franciscana* whole genome assembly, a complete set of ten *Hox* genes (*lab*, *pb*, *Hox3*, *Dfd*, *Scr*, *ftz*, *Antp*, *Ubx*, *abd-A*, and *Abd-B*) were identified (Table S13). All the *Hox* genes were located on chromosome 14, at positions between 22,007,211 and 34,940,254 in two subclusters (Table S13). In detail, *lab* and *pb* genes constituted the first subcluster while the remaining eight *Hox* genes constituted the second subcluster, and they contained a distance of ∼11.7 Mb between them (Table S13). Transcription of the *A. franciscana ftz* gene was proceeded in opposite direction, while all the other *Hox* genes were transcribed in the same direction (Table S13).

The 10 *Hox* genes were identified to contain a 63 amino acid-long homeodomain based on the Simple Modular Architecture Research Tool (SMART) (Table S13; Fig. S8A). Multiple alignments of amino acid sequences in the homeodomain show the relative amino acid frequencies and conserved amino acid positions (Table S13; Fig. S8B). Construction of a phylogenetic tree using the homeodomain amino acid sequences of the *Hox* genes from four species, including *A. franciscana*, *D. pulex*, *D. magna*, and *Drosophila melanogaster*, identified that most (eight out of ten) *Hox* genes formed a monophyletic cluster with their orthologs, while *Hox3* and *ftz* genes showed non-monophyly (Fig. S8C).

### 2.7 Homeobox gene expression profiles by developmental stages

The RNA expression profiles of the ten *Hox* genes were analyzed using RNA-Seq transcriptome sequencing in six developmental stages. In the early gastrula (0 h) stage, the *lab*, *Dfd*, *Scr*, *ftz*, *Antp*, and *Abd-B* genes were expressed, while *lab*, *Dfd*, and *Scr* genes were expressed in the late gastrula (12 h) stage (Fig. S9). In the early nauplius (16 h) and nauplius (24 h) stages, the *lab*, *pb*, *Dfd*, *Scr*, *ftz*, *Antp*, and *Abd-B* genes were expressed (Fig. S9). In the juvenile stage, the most diverse *Hox* genes were expressed (*lab*, *pb*, *Dfd*, *Scr*, *ftz*, *Antp*, *Ubx*, and *Abd-B*), yet the fewest number of genes were expressed in the adult stage, with only *lab* and *ftz* being expressed (Fig. S9). Two *Hox* genes (*Hox3* and *abd-A*) were not expressed across any of the developmental stages examined in this study, whereas *lab* gene showed relatively high levels of expression in all analyzed stages (Fig. S9).

### 2.8 Genomic organization of homeobox genes in arthropods

**Fig. 5:**
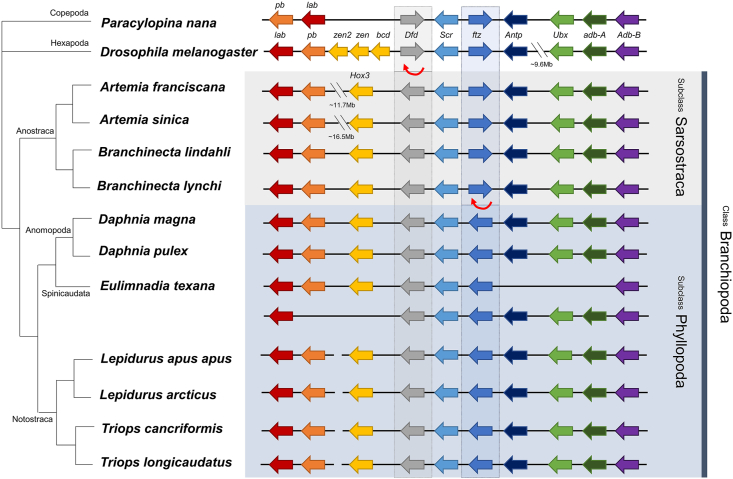
Comparison of genomic organization of homeobox (*Hox*) genes in arthropods. Thin red arrows below genes indicate putative inversion sites. (For interpretation of the references to color in this figure legend, the reader is referred to the Web version of this article.)

The genomic organization of the *Hox* genes was compared within the 13 arthropod species, including 11 Branchiopoda species across four orders, one Hexapoda (*D. melanogaster*), and one Copepoda (*Paracylopina nana*) (Table S14). Taxonomically, the class Branchiopoda is largely divided into two subclasses. One is Sarsostraca, which includes the order Anostraca that contains *Artemia*, while the other is Phyllopoda, which encompasses the rest of the branchiopods, apart from Anostraca. In the 11 species of Branchiopoda analyzed in this study, the *Dfd* gene had the inverse orientation compared to the one found in *D. melanogaster* and *P. nana* (Fig. 5). In addition, four species of Anostraca had the same orientation of the *ftz* gene as that found in *D. melanogaster* and *P. nana*, however, it was inverted in the Phyllopoda (Fig. 5). The order and orientation of all *Hox* genes were consistent in four anostracans (*i.e.*, *Artemia* and *Branchinecta*), thereby showing synteny conservation (Fig. 5).

## 3 Discussion

### 3.1 Improvement of genome quality and genomic characteristics

In this study, chromosome-level genome assembly of *A. franciscana* was constructed by combining various sequencing technologies. The *A. franciscana* genome had previously been reported by De Vos et al. [16] and Bett et al. [28]; however, this current study showed much-improved assembly statistics. This final genome assembly yielded a total length of ∼1.27 Gb with a scaffold N50 of 45.3 Mb, while the previous versions of the *A. franciscana* genome assemblies had a total size of 850 Mb with a scaffold N50 length of 112 kb [16] and a total size of ∼1.13 Gb with a scaffold N50 of 43.1 Mb [28]. Moreover, 21 scaffolds above 10 Mb in length were assigned to chromosome-level scaffolds in this study, indicating that 81 % of the assembly placed in the chromosomal super-scaffolds. Since it has been confirmed through previous karyotype studies [56,57] and linkage map results [25,26] that *A. franciscana* has 2n = 42 (or n = 21) chromosomes, these 21 super-scaffolds are assumed to be chromosomes. The complete BUSCO value was 91.0 %, which was significantly increased compared with the 63.3 % and 88.5 % completeness of the previous genome assembies [16,28]. All of these results demonstrate that the current genome assembly is more complete and highly contiguous, while also emphasizing the need to use a variety of the latest approaches to provide reference-quality genome assemblies. Meanwhile, the duplicated BUSCO value was somewhat high (5.9 %), and the duplication was also represented in the Merqury plot as a two-copy k-mer (blue peak). This may explain the slightly larger size of our assembly, which means there is still room for improvement for a perfect assembly.

The genome assembly of *A. franciscana* was compared with *A. sinica*, which resulted in a high syntenic correlation. It should be noted that this genome was assembled by reference-guided scaffolding based on the *A. sinica* genome and therefore may not reflect the actual structural variation of the genome between two species. This may be the reason why some SLAF-Seq markers of *A. franciscana* [26] were placed on different chromosomes in the blast results. However, before ordering and orienting the contigs of the genome by RagTag, initial scaffolding was performed with 10× linked-reads to N50 of >1 Mb. Therefore, it is expected that more reliable assembly results were obtained compared to using unscaffolded contigs. For further explanation, we extracted the RagTag scaffolded and contig-level split Z chromosome (chromosome 1) and aligned them to the SLAF marker-based assembly [28] (Fig. S10). This confirmed that two versions of chromosome 1 in this study were ordered in a similar pattern regardless of RagTag scaffolding. Furthermore, the structural integrity of the genome assembly was also confirmed by Hi-C. In the Hi-C heatmap, the interaction signals were observed across some chromosomes. These signals are probably considered ectopic interactions, indicating that these regions are located in close proximity in the tertiary structure of chromatin and physically interact with each other. Interestingly, the interactions were found between chromosome 1 (the sex chromosome) and chromosome 14 (where the *Hox* genes are located), two important chromosomes that contain genes associated with developmental processes. Previous studies in fruit fly [58] and silkworm [59] have found that joint regulation of sex determination genes and *Hox* genes controls sexual dimorphism. This suggests that, in *A. franciscana*, these interactions may play a critical role in the expression or regulation of development-related genes, sometimes influencing the determination of specific sexual differences or traits.

The present *A. franciscana* genome contained a high repeat content of 67.26 %, with TEs comprising 64.73 %, which was more abundant than in the previously reported *A. franciscana* (58 %) [16] and other branchiopod genomes (8.21–51.69 %) [60]. The highest repeat proportion in the *A. franciscana* genome is thought to be related to the relatively large genome size in Branchiopoda since repeat contents usually tend to increase with genome size [[61], [62], [63]]. Moreover, additional repeats could have been found by formulating a more contiguous chromosome-level assembly [62]. Among the TEs, rolling-circle transposons (Helitrons) accounted for 8.81 %, which represented a remarkably large proportion of the genome. Helitrons are usually known to be relevant to abiotic stresses (drought, light, and heat) in Arabidopsis [64] and regulation of heat shock responses in *Caenorhabditis elegans* [65], thereby suggesting that extreme habitat environments may have affected the proportion of Helitrons.

In the gene annotation result, 26,923 protein-coding genes were predicted by the EVM pipeline, which was comparable to other crustaceans that mainly had between 14,000 and 30,000 genes (https://www.ncbi.nlm.nih.gov/data-hub/genome/?taxon=6657, accessed on April 2023). Gene family gain-and-loss analysis was conducted on 14 crustacean species, including *A. franciscana*, and GO enrichment analysis was performed on the expanded gene families. The results showed that muscle cytoskeleton-related gene families were highly enriched in *A. franciscana*. Among all arthropods, crustaceans display a wide variety of limb types [66,67]. Muscle genes in crustaceans and *Artemia* have been relatively less studied [67,68]; however, comparisons of naupliar muscle developmental patterns between dendrobranchiate shrimp and *Artemia* showed different anatomical origins of extrinsic limb muscles, with overall pattern variation [69]. *Artemia* possess 11 pairs of swimming legs, which constantly move for locomotion, breathing, feeding, and osmoregulation [70], while further studies should be performed to determine whether these are also associated with *A. franciscana*-specific physiological properties.

### 3.2 Identification of sex chromosome and candidate sex-determining regions

This study assembled 20 autosomes and one large sex chromosome (chromosome 1, ∼64.0 Mb in length). We identified homologous regions at both ends of the chromosome 1, which thought to be shared in Z and W chromosomes (PARs), with the heterologous fragments of the W and Z chromosomes located between the PARs. Since the young strata of the W chromosome have not yet been fully degenerated [30], it is likely that the W chromosomal contigs were assembled together within the Z chromosome. Therefore, we manually removed five W contigs from the Z chromosome on the basis of the female and male WGS reads coverage ratios. The non-recombining regions in the *A. franciscana* that interleaved by the W fragments exist in smaller sizes than in mammalian species with the XY system, as in the findings in *A. sinica*, and seem to correspond to stratum 1a (S1a) [27]. The regions where only Z chromosome sequences were assembled without the intercalated W fragments appear to be highly differentiated regions that correspond to stratum 0 (S0) in the *A. sinica* genome [27]. Two scenarios are possible for the size of the sex chromosomes in *A. franciscana*: 1) Z and W chromosomes are different sizes, with Z being larger than W; 2) Z and W chromosomes are similar sizes. Karyotype research on *A. franciscana* reported that heteromorphic pairs consist of the longest (Z) and the shortest (W) chromosomes [56], although in another study, heteromorphy was not observed [57]. Thus, additional studies are needed to confirm whether the sex chromosomes are heteromorphic by assembling the W chromosome separately, although this was not successful in this study.

If the WGS mapping results of the female and male *A. franciscana* helped to identify the ZW sex chromosome and strata, the WTS mapping results could also be used in the analysis of the female and male expressions. There was no significant difference in mapping coverage depth between females and males across overall sex chromosome regions, which is thought to be due to dosage compensation by the Z chromosome [27,30]. In the W chromosomal fragments, several regions had more than two-fold differences in the mapping coverage depths in the female and male RNA-seq data, and the genes positioned in those regions included those known to be related to female functions or sex chromosomes. The ‘10 kDa heat shock protein, mitochondrial’ and ‘60 kDa heat shock protein, mitochondrial’ genes were located on the same chromosome in eukaryotes but had orientations that were in opposite directions; however, they remained functionally correlated by sharing a promotor [71]. These genes appear to be related to pregnancy and in particular the ‘60 kDa heat shock protein, mitochondrial’ functions, such as synthesizing progesterone and promoting blastocyte development [[71], [72], [73], [74], [75]]. Retrotransposons ‘Transposon Ty3-I Gag-Pol polyprotein’ and ‘RNA-directed DNA polymerase from mobile element jockey-like’ may play roles in sex chromosome differentiation and the translocation of sex-determination regions [76,77]. Subsequently, we designed PCR markers for 23 sex-determining genes located on the three W chromosomal contigs. Validation PCR using female and male DNA showed that at least nine genes, including the ‘60 kDa heat shock protein, mitochondrial’, were located on the W chromosome, as they were amplified only in the female. This partly verified and supported the bioinformatic results with molecular evidence by PCR, demonstrating that three of the five W contigs are indeed W chromosome fragments, and illustrated their utility as sex discrimination markers in *A. franciscana*.

On the tentative Z chromosome-specific region, two copies of the ‘chaoptin’ gene showed a mapping coverage depth that was more than twice as high in males. This phototransduction gene is known to be associated with female/male sexual dimorphisms of the lateral eyes in Ostracoda, which has the XX/XO sex-determination system [78]. Sexual dimorphism of eye size has also been observed in *A. franciscana* [79], although the genes involved remain unknown. This study suggests that the ‘chaoptin’ gene may be involved in sexual dimorphism of eye size or possibly even sex determination in *A. franciscana*. Species that are easy to raise and for which genome sequences and embryonic developmental data are available are promising as new model organisms for crustaceans [80]. *Artemia* has many advantages as a model organism in studying ZW sex-determination systems because of its ease of rearing, short life cycle, and the existence of two reproductive modes (sexual and asexual) [25,27,30,33]. The near-complete genome assembly of female *A. franciscana* produced in this study allowed the identification of the ZW chromosome regions and provided suggestions for sex-determination candidate genes.

### 3.3 Complete homeobox gene organizations

Based on the high-quality genome assembly, a total of ten *Hox* genes were completely identified in *A. franciscana*, with the order and directional information, and found to be located on a single chromosome: chromosome 14. A recent paper on the *Hox* genes in branchiopods analyzed that the *A. franciscana Hox* genes were located in four contigs [54], which might be due to the use of genome assembly based on short-read sequencing data. Even in recently published high-quality *A. franciscana* genome [28], the entire *Hox* gene cluster has not been identified. This study reveals that the *Hox* cluster in *A. franciscana* is divided into two subclusters, with a large distance of ∼11.7 Mb between *pb* and *Hox3*, and a gap of ∼500 kb between *abd-A* and *Abd-B*; therefore, it would have been difficult to detect these gaps using a short read-based genome assembly. Unlike in the *D. melanogaster Hox* cluster, which is split into two subclusters—the Antennapedia complex (ANT-C) and Bithorax complex (BX-C)—with ∼9.6 Mb between *Antp* and *Ubx* [42,81], the *Hox* cluster in the *Artemia* species has different subclusters. The *lab* and *pb* genes form a subcluster, the anterior paralogy group (APG), and the remaining *Hox* genes form another subcluster, known as the central/posterior paralogy group (CPPG) (terms referred from Nicolini et al. [54]). These subgroups were also present in the Notostraca species but located on two separate scaffolds [54]. These results seem to be evidence that there were changes in the *Hox* cluster arrangements in at least some branchiopods, which again underlines the importance of high-quality genomes for future *Hox* gene analyses.

The result of constructing the phylogenetic tree for the homeodomain sequences of *A. franciscana* with those of *D. melanogaster* and two *Daphnia* species showed that nearly every *Hox* gene generally formed monophyly, with the exceptions being *Hox3* and *ftz*. Non-monophyly of these genes is consistent with the previous study [54], thereby confirming the sequence divergences in the *Hox3* and *ftz* genes. The developmental roles of these two genes are known to be significantly changed in arthropods [36,82,83]. In insect lineages, including in fruit flies, the *Hox3* homologs—zerknüllt (*zen*) and bicoid (*bcd*)—are expressed in the extraembryonic ectoderm and the anterior blastoderm, respectively, and *ftz* is a pair-rule segmentation gene expressed in the early embryo. However, all of these genes have lost their *Hox*-like expression patterns along the A–P axis [36,82,[84], [85], [86], [87]]. Conversely, these genes retain *Hox*-like functions in mites, centipedes, water fleas, and amphipods [36,83,87,88]. In terms of *Artemia*, *ftz* in *A. salina* has lost the *Hox*-like function and gained a role in the central nervous system [47,89]. Although both belong to Branchiopoda, the results indicating that the *ftz* function was lost in *Artemia* but remained in *Daphnia* were somewhat confusing. The expression pattern of *Hox3* in *Artemia* has not yet been identified. Additionally, even RNA-Seq analysis according to the *A. franciscana* developmental stages did not show any *Hox3* expression among any of the developmental stages analyzed. The sequence divergences of *Hox3* and *ftz* in *A. franciscana* were quite high in this analysis, which may reflect dramatic functional changes by these genes in *Artemia* from *Drosophila* and *Daphnia*. The role of the *Hox3* and *ftz* genes in *A. franciscana* seems to require further investigation using a method such as *in situ* hybridization.

The *Hox* gene organization in *A. franciscana* was genomically compared to 12 other arthropod species. Compared to *Drosophila* and Copepoda, the transcriptional orientation of the *Dfd* gene was inverted in all four orders of the Branchiopoda included in this analysis: Anostraca, Anomopoda, Spinicaudata, and Notostraca. In addition, within Branchiopoda, the subclasses Sarsostraca (including Anostraca) and Phyllopoda (including Anomopoda, Spinicaudata, and Notostraca) presented opposite transcriptional directions for the *ftz* gene. All *Hox* genes in an ancestral arthropod are presumed to be arranged in the same transcriptional orientation [42]; thus, it can be inferred that the *Hox* gene in *Daphnia* retained the same orientation as the ancestral arthropod. The direction of the *ftz* gene in *Artemia* was inverted compared to the ancestral arthropod, as in *Drosophila* and Copepoda. Considering the remarkable functional changes in the *ftz* gene in *Drosophila* and *Artemia*, it is suggested that this inversion of the *ftz* gene may be related to the loss of the *Hox*-like expression. However, since there are only a limited number of species whose expression has been studied in Phyllopoda (only in *Daphnia*), further studies on other taxa, such as Notostraca, are required to provide further conclusions. The mitochondrial genomes of Anostraca and Phyllopoda clades were also shown to have different gene orders, with Phyllopoda presenting a more ancestral pattern [90,91]. It appears that these groups, which diverged a long time ago (385.23 million years ago (MYA), refer to the TimeTree result), have evolved independently in both the rapidly evolving mitochondrial genes and the *Hox* genes.

## 4 Conclusions

In this study, the high-quality chromosome-level genome assembly of female *A. franciscana* was achieved by combining Illumina short-reads, PacBio long-reads, 10× linked-reads, and Hi-C sequencing. Through this genome information, the female-specific W contigs and the Z-specific region on the *A. franciscana* sex chromosome were identified, with suggestions of W-specific markers and candidate sex-determination genes. In addition, the entire structures of the ten canonical *Hox* genes in *A. franciscana* were first identified and their syntenic evolution was inferred through comparison within arthropods. It is necessary to study the structural and functional features and gene regulation mechanisms in more depth by linking the discovery of sex chromosomes and *Hox* genes with the Hi-C interactions that appears between chromosomes. This study provides a useful reference for genomic and genetic research on *A. franciscana* and will help further studies to deeply understand the evolutionary and developmental characteristics of *A. franciscana*.

## 5 Limitations of the study

The first limitation of this study is that the W sex chromosomes were not completely assembled. There are biological and bioinformatical challenges to assembling the reference genome [92]. The assembly of sex chromosomes is much more difficult than autosomes due to the complex nature of sex-determining regions and the sequencing coverage is about 50 % less than autosomes [93]. Nevertheless, this study almost successfully assembled one large Z sex chromosome additional five W chromosomal contigs for female *A. franciscana*. The second limitation is that approximately 19 % (∼240 Mb) of the total assembly length (∼1.27 Gb) still remains unanchored to chromosomes. This may be due to the reference-guided assembly using the male genome of a congeneric species, *A. sinica*. However, the assembly in this study serves as a meaningful reference genome for *A. franciscana* on its own and will be improved with more advanced tools in the future. The third limitation is that sex determination candidates and *Hox* genes were identified only through bioinformatic analysis not functional study. We developed W chromosome-specific markers, which confirmed nine sex determination candidate genes located on the W chromosome by PCR, thereby resolving these analytical limitations to some extent. Further studies on those genes using RNAi or CRISPR could reveal their functions in *A. franciscana* more accurately.

## 6 Materials and methods

### 6.1 Resource availability

#### 6.1.1 Lead contact

Further information and requests for resources and reagents should be directed to and will be fulfilled by the lead contact, Hyun Park (hpark@korea.ac.kr).

#### 6.1.2 Materials availability

This study did not generate new unique reagents.

#### 6.1.3 Data availability statement

The *Artemia franciscana* genome assembly and annotation data have been deposited at DDBJ/ENA/GenBank under the accession JAVRJZ000000000 and BioProject number PRJNA991266. Accession numbers are listed in the key resources table. This paper does not report original code. Any additional information required to reanalyze the data reported in this paper is available from the lead contact upon request.

### 6.2 Experimental model and study participant details

#### 6.2.1 Animal culture

Commercial *Artemia* cysts (INVE Technologies NV, Dendermonde, Belgium) from the Great Salt Lake were hatched into nauplii in 30 g/L salt water by aeration for ∼1 day at 25 °C. The culture was maintained by feeding live green algae, *Tetraselmis* sp., every other day.

### 6.3 Method details

#### 6.3.1 Species identification

A female individual incubating eggs was cultured separately, and the genomic DNA was extracted from the progeny using a QIAamp DNA Micro kit (QIAGEN, Hilden, Germany) for molecular species identification. Amplification was conducted using the polymerase chain reaction (PCR) on the mitochondrial cytochrome *c* oxidase I (*COI*) gene, and it was identified as *A. franciscana* by the Basic Local Alignment Search Tool (BLAST) search at the National Center for Biotechnology Information (NCBI). The progenies from the maternal lineage were used for the subsequent experiments.

#### 6.3.2 Long-read sequencing and assembly

For long-read sequencing, 14 female adults were used from the culture to extract high-molecular-weight (HMW) DNA via the phenol/chloroform method. The quality and quantity of the DNA were checked using a Fragment Analyzer (Agilent Technologies, Santa Clara, CA, USA) and a Qubit fluorometer (Invitrogen, Life Technologies, Carlsbad, CA, USA). The HMW DNA was used to prepare 20 kb size-selected PacBio Sequel libraries, according to the manufacturer's protocol (Pacific Biosciences, Menlo Park, CA, USA). In detail, the SMRT sequencing library was constructed using SMRTbell template prep kit 1.0, and the SMRTbell-polymerase complex was generated by Sequel binding kit 3.0 (Pacific Biosciences, Menlo Park, CA, USA). The complex was loaded onto two SMRT cells 1M v3 and sequenced using a Sequel sequencing kit 3.0 (Pacific Biosciences, Menlo Park, CA, USA), with a 600-min movie time per cell. *De novo* genome assembly was performed by Peregrine [94], using default options. Purge Haplotigs pipeline [95] was conducted to identify and deduplicate haplotigs in the assembly.

#### 6.3.3 10× linked-read sequencing and scaffolding

The 10× linked-read sequencing library was prepared using one female individual following the Chromium Genome Reagent Kits Version 2 User Guide (PN-120258) (10× Genomics, Pleasanton, CA, USA). The 10× barcoded gel beads were combined with ∼1.25 ng of HMW DNA and partitioning oil to generate gel bead-in-emulsions (GEMs) in the microfluidic Genome chip (PN-120257), using the 10× Genomics Chromium Controller. Then, the GEMs were isothermally incubated to produce barcoded amplicons and cleaned up by bead-based methods, followed by Illumina library construction. Library yield and fragment size were measured using a Qubit dsDNA HS Assay Kit (Invitrogen, Life Technologies, CA, USA) and Agilent 2100 Bioanalyzer High Sensitivity DNA chip (Agilent Technologies, Santa Clara, CA, USA). The library was sequenced on a NovaSeq 6000 platform using the 2 × 150 bp protocol (Illumina, San Diego, CA, USA). The 10× linked-read data were used to correct misassembly and scaffold the draft assembly using ARCS + LINKS + Tigmint pipeline [[96], [97], [98]] with the following options: t = 8 c = 3 l = 3 a = 0.7 s = 90.

#### 6.3.4 Polishing the genome assembly

The draft genome assembly was polished by Pilon v1.23 [99] to improve its accuracy, using a BAM file generated by BWA [100] along with the short-read data produced in the previous genome survey sequencing [55].

#### 6.3.5 Hi-C sequencing and chromosome-level scaffolding

The Dovetail™ Hi-C library was constructed using the frozen 66 female samples, following the Dovetail™ Hi-C kit manual (Dovetail Genomics, Scotts Valley, CA, USA). The Hi-C library was sequenced on an Illumina NovaSeq 6000 platform for a 2 × 150 bp paired-end run. The resulting Hi-C reads were used for super-scaffolding the polished draft assembly. A candidate genome assembly was generated using Juicer v1.5.7 [101] and the 3D *de novo* assembly (3D-DNA) [102] pipelines, followed by manual curation with Juicebox v1.5 [103]; however, the scaffolding did not work well. Thus, the draft assembly was further scaffolded using the RagTag scaffold tool [104], with the *A. sinica* genome assembly (https://doi.org/10.15479/AT:ISTA:11653, accessed on September 2022) [27] used as a reference. Afterward, sex chromosome (chromosome 1) was separated into Z and W-specific contigs by comparing differences in mapping coverage ratio of female and male WGS reads [105,106], using the threshold of |log_2_ (M:F)| > 1. Five W contigs were identified and manually extracted from the Z chromosome with reference to the position information of the contigs in the chromosome 1 derived from the RagTag scaffolding AGP file. The Hi-C reads were aligned to the RagTag assembly using Juicer v1.5.7 [101]. The Hi-C contact map was visualized using inter_30. hic file from Juicebox v1.5 [103]. The genome assembly of *A. franciscana* was finalized by filtering out small scaffolds less than 1 kb in length. The overall bioinformatics workflow of *A. franciscana* genome assembly is summarized in Fig. 1A.

#### 6.3.6 Quality assessments

To assess the completeness of the assembly, BUSCO v5.6.1 [107] was performed with Augustus gene predictor [108] in genome assessment mode using the Eukaryota_odb10 dataset. The assembly statistics were calculated using the script ‘assemblathon_stats.pl’ (http://korflab.ucdavis.edu/datasets/assemblathon/assemblathon2/basic_metrics/assemblathon_stats.pl, accessed on September 2022). The contiguity of the assembly was evaluated with an N50 value, which is defined as the length of the shortest contig or scaffold that accounts for 50 % of the total genome length. Additionally, the quality value (QV), k-mer error rate, and k-mer completeness were evaluated by Merqury v1.3 [109] with the k-mer of 19.

#### 6.3.7 Comparative genomics analysis

To compare genome sequences at the chromosome level, the NUCmer pipeline was implemented on the MUMmer v4.02 [110]. To clarify the chromosome comparison, long scaffolds, regarded as chromosome-level (>10 Mb), were extracted from genome assemblies, and the remaining small scaffolds and contigs were removed. Accordingly, the 21 long scaffolds were aligned to the 21 chromosomes from the *A. sinica* genome assembly [27], using the options: -mum -c 1000 -g 10000. The output coordinates file from NUCmer was visualized in a circular layout using Circos software package [111].

#### 6.3.8 Transcriptome sequencing

The six developmental stages of *A. franciscana* were selected for transcriptome sequencing, as follows: 0 h (gastrula), 12 h (late gastrula), 16 h (early nauplius), 24 h (nauplius), juvenile, and adult. The total RNA for each sample was isolated using TRIzol Reagent (Ambion, Austin, TX, USA), according to the manufacturer's protocol. The RNA was qualified and quantified using a 2100 Bioanalyzer (Agilent Technologies, Santa Clara, CA, USA) and Qubit 2.0 fluorometer (Invitrogen, Life Technologies, CA, USA), respectively. Illumina RNA-Seq libraries were prepared using a Truseq Stranded mRNA Prep kit (Illumina, San Diego, CA, USA), in accordance with the manufacturer's protocol, and sequenced on the Illumina HiSeq platform. Additionally, the RNA-Seq and Iso-Seq reads for *A. franciscana* females and males generated by the previous sex-biased transcriptome study [32] were also used for gene annotations.

#### 6.3.9 Repeat analysis and masking

A *de novo* repeat library was produced using RepeatModeler v1.0.3 [112] with default settings. All repeats detected by RepeatModeler, except for transposons, were searched against the UniProt/SwissProt database [113]. With the merged repeat library, repetitive elements were masked using RepeatMasker v4.0.9 (https://www.repeatmasker.org/, accessed on December 2022) [114]. The scripts ‘calcDivergenceFromAlign.pl’ and ‘createRepeatLandscape.pl’ were used to calculate the Kimura divergence values [115] for each alignment and to plot the interspersed repeat landscape.

#### 6.3.10 Gene prediction and functional annotation

Gene structural annotation was conducted using EVM v1.1.1 [116], which combines multiple types of evidence for gene prediction. Initially, *de novo* transcriptome assembly was performed using Trinity v2.8.5 [117] with RNA-Seq data generated by gender [32] and developmental stages. Trinity-reconstructed transcripts and two kinds of Iso-Seq data, produced in the previous transcriptome study by pooling female and male [32], and downloaded from NCBI sequence read archive (SRA) (SRR12358297) [118], were used as inputs for the Program to Assemble Spliced Alignments (PASA) pipeline v2.5.1 [119], to generate transcript evidence. The transcripts were cleaned by seqclean utility, and then, mapped and aligned to the genome using BLAT v35 [120], GMAP v2021-08-25 [121], and minimap2 v2.24 (r1122) [122] aligners. Subsequently, *ab initio* gene prediction was conducted with the repeat masked genome assembly using GeneMark-ES v4.68 [123]. Then, protein hints were generated with Arthropoda protein sequences in the SwissProt database [113] using ProtHint v2.6.0 [124]. The hints files were used to make protein-based evidence using GeneMark-EP + v4.68 [124] and for *ab initio* gene predictions using Augustus v3.4.0 [108]. EVM integrated all gene models with weight values of ABINITIO_PREDICTION 1, PROTEIN 50, and TRANSCRIPT 50 to make consensus gene structures. Finally, consensus gene prediction was updated using the PASA pipeline [119] by adding untranslated regions (UTRs) and alternatively spliced isoforms based on the assembled RNA-Seq and Iso-Seq data. The completeness of the annotations was assessed by BUSCO v4.1.2 [107], in transcriptome mode, along with the Eukaryota_odb10 lineage dataset.

The predicted genes were annotated by aligning to the NCBI non-redundant protein (nr) database [125] using BLASTP v2.9.0 [126], with an E-value cutoff value of 1e-5. InterProScan v5.44.79 [127] was used to predict protein function with protein sequences translated from transcripts. GO terms were assigned to the annotated sequences using the Blast2GO [128] module implemented in OmicsBox v1.3.11 [129]. KEGG pathway annotation was conducted using the KEGG Automatic Annotation Server (KAAS) [130] and KEGG Mapper [131]. Additionally, Trinotate v3.2.0 [132] was used for comprehensive functional annotation of transcriptome sequences. Briefly, predicted coding regions were extracted using TransDecoder v5.5.0 (https://github.com/TransDecoder/TransDecoder, accessed on January 2023) followed by sequence homologies search using BLAST [126] against UniProt/SwissProt database [113], protein domain identification using HMMER [133] via Pfam database [134], protein signal peptides prediction using SignalP v5.0 [135], and transmembrane domain prediction using TMHMM v2.0 [136].

#### 6.3.11 Gene family identification and phylogenetic analysis

The protein sequences of 13 crustacean species were downloaded from public databases for the orthologous analysis (Table S6). The orthogroups across 14 crustaceans were found based on their protein sequence similarity using OrthoFinder 2 [137] with default parameters. The phylogenetic tree was constructed from the concatenated protein sequence alignments of the 113 SCOGs shared by 14 crustaceans using the maximum-likelihood (ML) method and the Jones-Taylor-Thornton (JTT) substitution model [138] in MEGA X software [139]. The divergence times were calibrated using the RelTime method [140,141], with the time estimates data from TimeTree 5 (https://timetree.org/, accessed on August 2023) [142]. The TimeTree was computed by setting minimum and maximum time boundaries [143] for the following three calibration constraints: (1) *A. franciscana* versus *Daphnia pulex* (365.1–491.7 MYA), (2) *A. franciscana* versus *Penaeus vannamei* (275.0–541.0 MYA), and (3) *P. vannamei* versus *Hyalella azteca* (169.1–388.2 MYA). The gene family gain-and-loss was analyzed using CAFE v4.2.1 [144] using the options -p 0.05 and -filter. Orthologous gene clusters among representative species of four orders within Branchiopoda were compared and visualized using OrthoVenn3 [145]. GO enrichment analysis using a Fisher's exact test (P-value <0.05) was performed via OmicsBox v3.0.30 [[128], [129]] on the gene families that were specifically expanded in *A. franciscana*.

#### 6.3.12 Whole genome sequencing by sex and reads mapping to chromosomes

DNA was extracted from one female and one male using the PCI method, followed by WGS on the Illumina Novaseq 6000 platform. The read pairs from whole genome sequencing were mapped to the genome assembly separately for females and males using BWA [100], with mapping quality ≥60. Similarly, each of the female and male RNA-Seq reads were aligned to the genome assembly using Bowtie2 [146]. Subsequently, SAM alignment files were converted to sorted BAM files using SAMtools v1.10 [147] and genome coverages were calculated by 100 k intervals using BEDtools v2.29.2 [148]. Data visualization was conducted by ggplot2 package [149] in R v4.1.3 [150], via RStudio v2022.12.0.353 [151]. To confirm the continuity of the genome assembly, SLAF-Seq markers obtained for *A. franciscana* from Han et al. [26] were BLASTed to the final genome assembly, and the number of markers that matched each LG and chromosome with ≥95 % identity was counted.

#### 6.3.13 PCR validation of W chromosome genes

The PCR primers for the 23 genes estimated to be located on the W chromosome were designed using primer3 [152] implemented in Geneious v9.1.2 (https://www.geneious.com). The primers were preferentially positioned in the intronic region flanking an exon of each gene, with the options for PCR product length of 300–500 bp, primer size of 18–22 mer, GC content of 20–80 %, and melting temperature of 58–63 °C. PCR amplification was performed in total reaction volume of 25 μL including 12.5 μL 2× EmeraldAmp GT PCR Master Mix (Takara Bio, Shiga, Japan), 0.5 μL genomic DNA for female and male, 0.5 μL (10 pmol/L) each forward and reverse primers, and 11 μL ddH_2_O. The PCR program was set an initial denaturation at 94 °C for 3 min, followed by 30 cycles of 95 °C for 15 s, 60 °C for 30 s, and 72 °C for 30 s, with a final extension of 72 °C for 10 min. The electrophoresis was conducted on 2 % agarose gels with NICSROgene™ Fluoro 100 bp+3K DNA ladder (Bionics, Seoul, Korea).

#### 6.3.14 Homeobox gene identification

The final genome assembly was used to identify the *Hox* gene cluster in *A. franciscana*. A local BLAST database was created with the final *A. franciscana* genome assembly. The protein sequences of the *Daphnia magna Hox* genes were downloaded from NCBI (GenBank accession no. MF497307-16) [53] and imported into BLAST, using the *A. franciscana* final assembly as a reference to determine which scaffold the *Hox* genes were located on and roughly estimate their positions in the scaffold. Subsequently, JBrowse [153] was established using the genome assembly FASTA and GFF annotation files to visualize the gene annotation tracks. Based on the scaffold ID and position information identified through BLAST, the start and stop positions and number of introns for each *Hox* gene were confirmed.

#### 6.3.15 Phylogenetic analysis of the homeobox domain

The homeodomains of the ten *Hox* genes in *A. franciscana* were identified using the SMART domain search tool (http://smart.embl-heidelberg.de/, accessed on February 2023) [154]. The conserved homeodomain sequences in *A. franciscana* were aligned by Geneious global alignment (Blosum 62 cost matrix with gap open penalty 12, gap extension penalty 3, and refinement iterations 2 parameters) in Geneious v9.1.2 (https://www.geneious.com), and the alignments were submitted to WebLogo3 (https://weblogo.threeplusone.com/create.cgi, accessed on February 2023) [155] to generate a graphical sequence logo.

The *Hox* gene datasets for three species, *D. pulex*, *D. magna*, and *Drosophila melanogaster*, were downloaded from online repositories (https://github.com/filonico/branchiopoda_Hox_ParaHox, accessed on February 2023) [54] and used in the phylogenetic analysis. A total of 42 amino acid sequences were aligned for *Hox* genes from four species, including *A. franciscana*, by Clustal W alignment (BLOSUM cost matrix with gap opening cost 9 and gap extend cost 0.1 options), using Geneious v9.1.2 (https://www.geneious.com). A phylogenetic tree of the homeodomain sequences was constructed for the four species using MEGA X software [139], via the ML method based on the JTT + I model [138].

#### 6.3.16 Homeobox gene expression analysis by developmental stages

The RNA-Seq reads of six developmental stages were mapped to the *A. franciscana Hox* gene nucleotide sequences using CLC Genomics Workbench v12.0.3 (QIAGEN, Aarhus, Denmark). The *Hox* gene expression levels, according to six developmental stages, were normalized by log 2 ratio of transcripts per million (TPM) values and displayed as a heatmap using MultiExperiment Viewer (MeV) v4.9.0 [156].

#### 6.3.17 Genomic comparison of homeobox genes in arthropods

A total of 13 arthropod species were used for comparative analysis of *Hox* genes (Table S13). The *Hox* genes in *D. melanogaster* were identified by searching with the JBrowse Genome viewer in FlyBase (FB2022_01 release) [157]. The nucleotide sequence of the *Hox* gene cluster region for *Paracylopina nana* was obtained from NCBI (GenBank accession no. KT345727.1) [158]. For the following ten branchiopod species, the genome assembly FASTA files were downloaded from NCBI: *A. sinica* (GCA_027921565.1) [27], *Branchinecta lindahli* (GCA_023053555.1) [159], *B. lynchi* (GCA_023053575.1) [160], *Daphnia magna* (GCA_020631705.2) [53], *D. pulex* (GCA_021134715.1), *Eulimnadia texana* (GCA_002872375.1) [51], *Lepidurus arcticus* (GCA_003724045.1) [161], *L. apus apus* (GCA_022832285.1) [60], *Triops cancriformis* (GCA_020615345.1) [162], and *T. longicaudatus* (GCA_022885665.1) [60]. The *Hox* gene orders and orientations were confirmed by conducting a tBLASTn on the *D. magna Hox* protein sequences [53], using the genome assemblies of these species.

**Key resources table**

**Table:** 

REAGENT or RESOURCE	SOURCE	IDENTIFIER
Deposited data		
*Artemia franciscana* female genome assembly	This paper	PRJNA991266
*A. franciscana* Illumina sequences	Jo et al. [55]	PRJNA449186
*A. franciscana* RNA-Seq and Iso-Seq (female, male)	Jo et al. [32]	SRR14598203–SRR14598205
*A. franciscana* Iso-Seq (150 psμ condition)	Lee et al. [118]	SRR12358297
*A. franciscana* SLAF-Seq markers	Han et al. [26]	N/A
*A. sinica* male genome assembly	Elkrewi et al. [27]	PRJNA848277; https://research-explorer.ista.ac.at/record/11653
*Daphnia magna Hox* genes	Kim et al. [53]	MF497307–16
Branchiopoda *Hox* genes	Nicolini et al. [53]	https://github.com/filonico/branchiopoda_Hox_ParaHox
*Drosophila melanogaster* genome assembly	Gramates et al. [157]	FlyBase: FB2022_01 release
*Paracylopina nana Hox* genes	Kim et al. [158]	KT345727.1
*Branchinecta lindahli* genome assembly	Kieran Blair et al. [159]	GCA_023053555.1
*B. lynchi* genome assembly	Kieran Blair et al. [160]	GCA_023053575.1
*Daphnia magna* genome assembly	Kim et al. [53]	GCA_020631705.2
*D. pulex* genome assembly	Arizona State University	GCA_021134715.1
*Eulimnadia texana* genome assembly	Baldwin-Brown et al. [51]	GCA_002872375.1
*Lepidurus arcticus* genome assembly	Savojardo et al. [161]	GCA_003724045.1
*L. apus apus* genome assembly	Luchetti et al. [60]	GCA_022832285.1
*Triops cancriformis* genome assembly	Orr [162]	GCA_020615345.1
*T. longicaudatus* genome assembly	Luchetti et al. [60]	GCA_022885665.1
Experimental models: Organisms/strains		
*Artemia franciscana*	This paper	N/A
Software and algorithms		
Peregrine	Chin and Khalak [94]	https://github.com/cschin/Peregrine
Purge Haplotigs	Roach et al. [95]	https://bitbucket.org/mroachawri/purge_haplotigs/src/master/
ARCS	Yeo et al. [98]	https://github.com/bcgsc/arcs
LINKS	Warren et al. [97]	https://github.com/bcgsc/LINKS
Tigmint	Jackman et al. [96]	https://github.com/bcgsc/tigmint
Pilon v1.23	Walker et al. [99]	https://github.com/broadinstitute/pilon
BWA	Li and Durbin [100]	https://github.com/lh3/bwa
RagTag	Alonge et al. [104]	https://github.com/malonge/RagTag
Juicer	Durand et al. [101]	https://github.com/aidenlab/juicer
Juicebox	Durand et al. [103]	https://github.com/aidenlab/Juicebox
BUSCO v4.1.2, v5.6.1	Manni et al. [107]	https://busco.ezlab.org
Merqury v1.3	Rhie et al. [109]	https://github.com/marbl/merqury
MUMmer v4.02	Marçais et al. [110]	https://github.com/mummer4/mummer
Circos	Krzywinski et al. [111]	http://circos.ca/
RepeatModeler v1.0.3	Hubley and Smit [112]	https://www.repeatmasker.org/RepeatModeler/
RepeatMasker v4.0.9	Smit et al. [114]	https://www.repeatmasker.org/RepeatMasker/
EVidenceModeler (EVM) v1.1.1	Haas et al. [116]	https://github.com/EVidenceModeler
Trinity v2.8.5	Grabherr et al. [117]	https://github.com/trinityrnaseq/trinityrnaseq
Program to Assemble Spliced Alignments (PASA) v2.5.1	Haas et al. [119]	https://github.com/PASApipeline/PASApipeline
BLAT v35	Kent [120]	https://genome.ucsc.edu/
GMAP v2021-08-25	Wu and Watanabe [121]	http://research-pub.gene.com/gmap/
Minimap2 v2.24 (r1122)	Li [122]	https://github.com/lh3/minimap2
GeneMark-ES v4.68	Lomsadze et al. [123]	http://exon.gatech.edu/genemark/index.html
ProtHint v2.6.0 and GeneMark-EP + v4.68	Brůna et al. [124]	https://github.com/gatech-genemark/GeneMark-EP-ProtHint-exp
Augustus v3.4.0	Stanke et al. [108]	https://github.com/Gaius-Augustus/Augustus
BLASTP v2.9.0	Altschul et al. [126]	https://blast.ncbi.nlm.nih.gov/Blast.cgi
InterProScan v5.44.79	Jones et al. [127]	https://www.ebi.ac.uk/interpro/about/interproscan/
OmicsBox v1.3.11 and v3.0.30	BioBam Bioinformatics [128,129]	https://www.biobam.com/omicsbox/
KEGG Automatic Annotation Server (KAAS)	Moriya et al. [130]	https://www.genome.jp/kegg/kaas/
KEGG Mapper	Kanehisa and Sato [131]	https://www.genome.jp/kegg/mapper/
Trinotate v3.2.0	Bryant et al. [132]	https://github.com/Trinotate/Trinotate
OrthoFinder 2	Emms and Kelly [137]	https://github.com/davidemms/OrthoFinder
MEGA X	Kumar et al. [138]	https://www.megasoftware.net/
TimeTree 5	Kumar et al. [142]	https://timetree.org/
CAFE v4.2.1	Han et al. [144]	https://github.com/hahnlab/CAFE
OrthoVenn3	Sun et al. [145]	https://orthovenn3.bioinfotoolkits.net/
Bowtie2	Langmead and Salzberg [146]	https://bowtie-bio.sourceforge.net/bowtie2/index.shtml
SAMtools v1.10	Danecek et al. [147]	https://github.com/samtools/samtools
BEDtools v2.29.2	Quinlan and Hall [148]	https://github.com/arq5x/bedtools2
ggplot2 package	Wickham [149]	https://ggplot2.tidyverse.org/
R v4.1.3	R Core Team [150]	https://www.r-project.org/
RStudio v2022.12.0.353	RStudio Team [151]	https://github.com/rstudio/rstudio
JBrowse	Diesh et al. [153]	https://github.com/GMOD/jbrowse
SMART	Letunic et al. [154]	http://smart.embl-heidelberg.de/
Geneious v9.1.2	Biomatters Ltd.	https://www.geneious.com/
WebLogo3	Crooks et al. [155]	https://weblogo.threeplusone.com/
CLC Genomics Workbench v12.0.3	QIAGEN	https://resources.qiagenbioinformatics.com/manuals/clcgenomicsworkbench/801/index.php
MultiExperiment Viewer (MeV) v4.9.0	Saeed et al. [156]	https://sourceforge.net/projects/mev-tm4/

## CRediT authorship contribution statement

**Euna Jo:** Writing – review & editing, Writing – original draft, Visualization, Validation, Software, Resources, Methodology, Investigation, Formal analysis, Data curation. **Minjoo Cho:** Writing – review & editing, Writing – original draft, Visualization, Validation, Methodology. **Soyun Choi:** Writing – review & editing, Methodology. **Seung Jae Lee:** Writing – review & editing, Visualization, Validation, Software, Methodology. **Eunkyung Choi:** Writing – review & editing, Software. **Jinmu Kim:** Writing – review & editing, Software. **Jang Yeon Kim:** Writing – review & editing, Software. **Sooyeon Kwon:** Writing – review & editing, Software. **Jun Hyuck Lee:** Writing – review & editing, Writing – original draft, Funding acquisition. **Hyun Park:** Writing – review & editing, Writing – original draft, Supervision, Funding acquisition, Conceptualization.

## Declaration of competing interest

The authors declare that they have no known competing financial interests or personal relationships that could have appeared to influence the work reported in this paper.
