# Supplementary material for: High-quality chromosome-level genome assembly of female Artemia franciscana reveals sex chromosome and Hox gene organization
Source: Heliyon. 2024 Sep 28;10(19):e38687. doi: 10.1016/j.heliyon.2024.e38687 (PMC11492255; doi:10.1016/j.heliyon.2024.e38687)
Supplement: Multimedia component 1 [file mmc1.docx]

**A B**


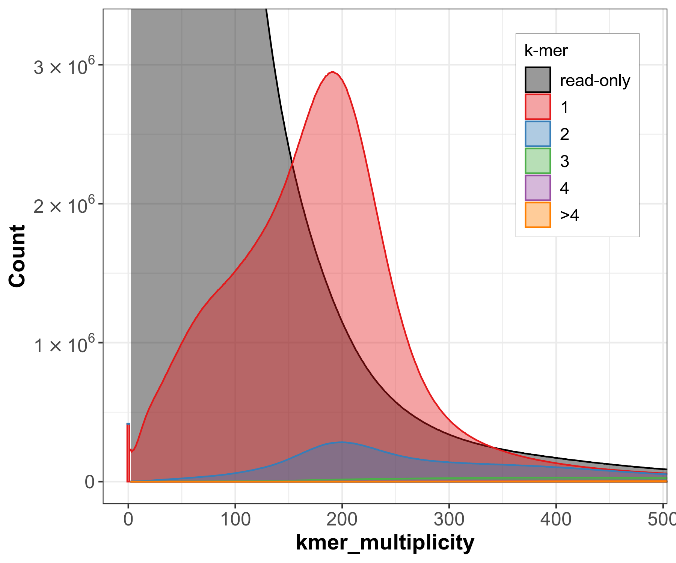

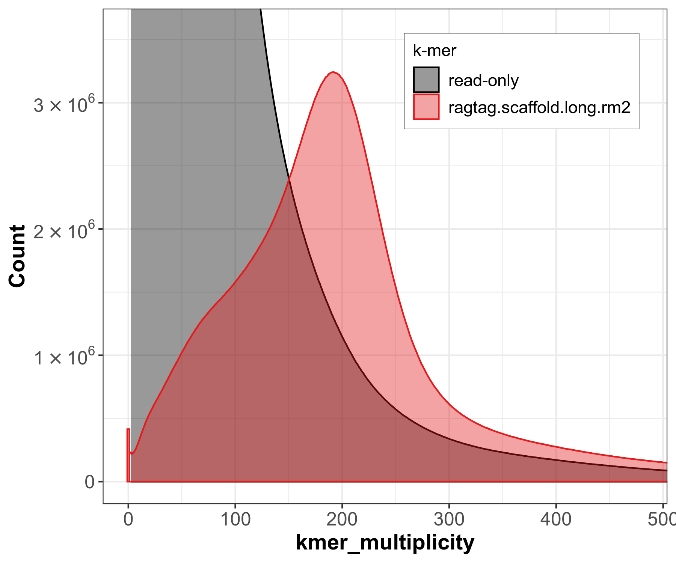


**Figure S1.** Merqury spectrum plots for *Artemia franciscana* genome assembly. **(A)** Copy number spectrum plot. **(B)** Assembly spectrum plot for evaluating K-mer completeness. Hybrid k-mer DB was built using Illumina short reads, 10x linked reads, and PacBio high-fidelity (HiFi) long reads. Read-only portions (grey) are k-mers that exist in the reads but not in the assembly, which tend to be larger when using HiFi reads.


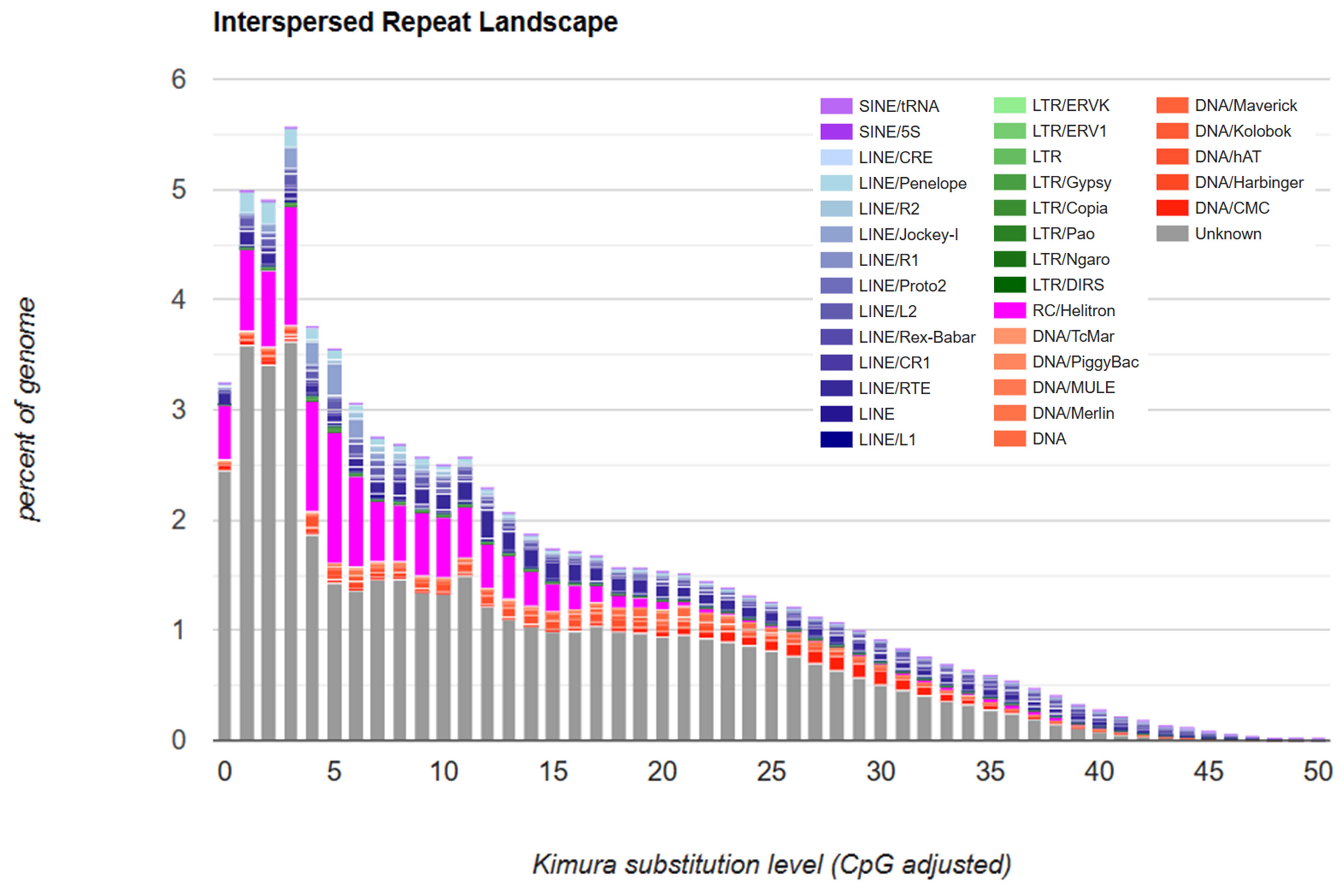


**Figure S2.** Kimura distance-based copy divergences of TEs in *Artemia franciscana* genome. Graphs represent genome coverage (Y-axis) for each type of TE and Kimura distances to their corresponding consensus sequence (X-axis, K-value from 0 to 50).





**Figure S3.** Gene Ontology (GO) annotations of the predicted genes of the *Artemia franciscana* genome. GO terms were classified into three main categories: biological process (BP, green), molecular function (MF, blue), and cellular component (CC, yellow). The horizontal axis indicates the number of genes in each class, and the vertical axis indicates the classes of the level 5 GO-annotation.


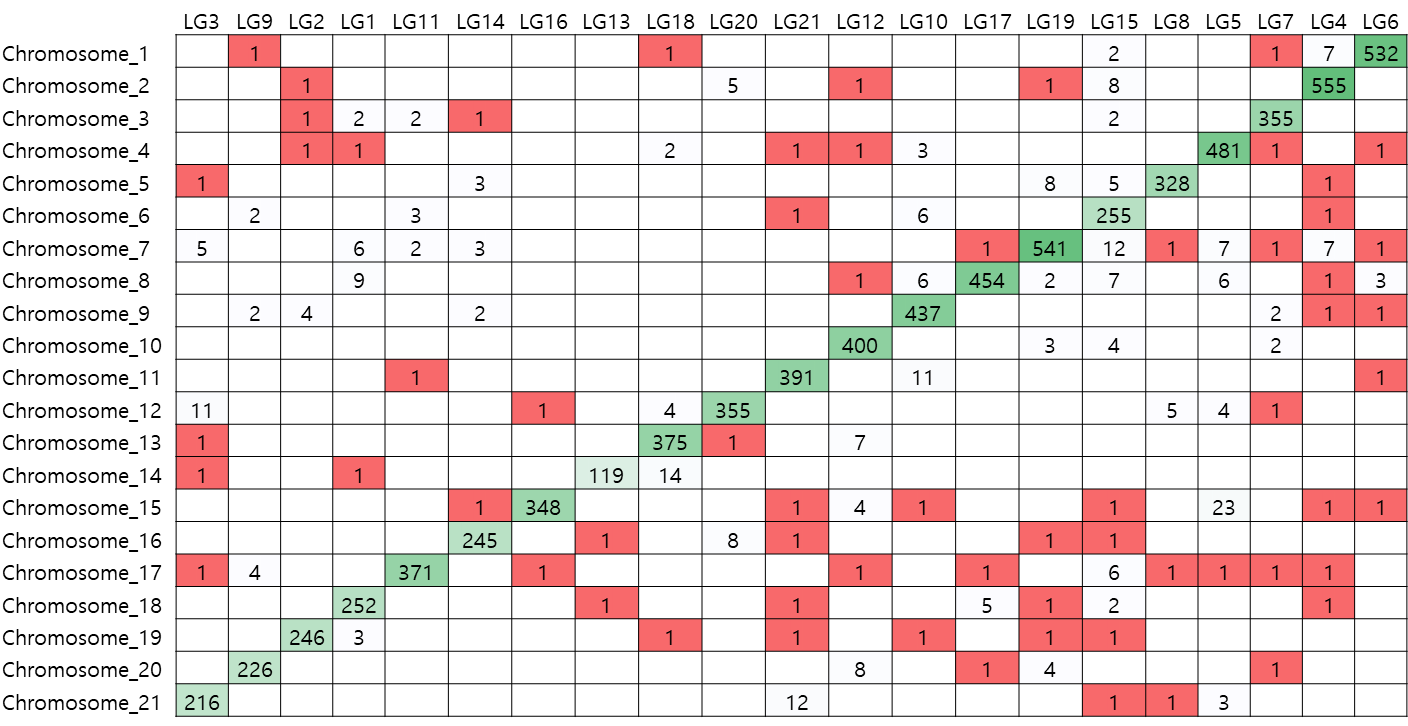


**Figure S4.** The number of SLAF markers in 21 linkage groups BLASTed to the final *Artemia franciscana* genome assembly. The green boxes mean the most markers matched, and the red boxes mean the fewest markers matched.


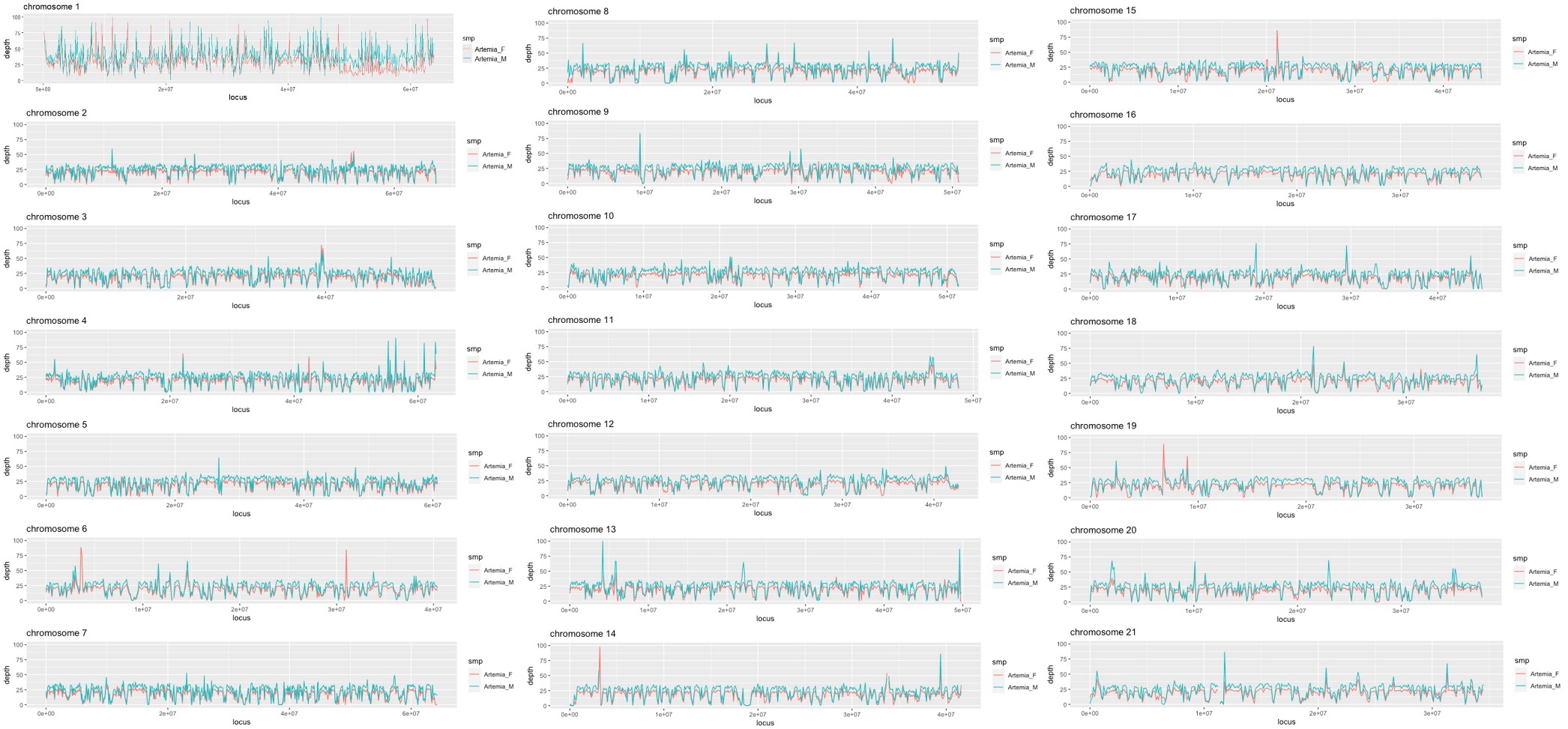


**Figure S5.** Coverage depths of female and male whole genome sequencing data to final 21 chromosomes of *Artemia franciscana*. Scarlet color plots represent female mapping reads, and mint color plots represent male mapping reads.


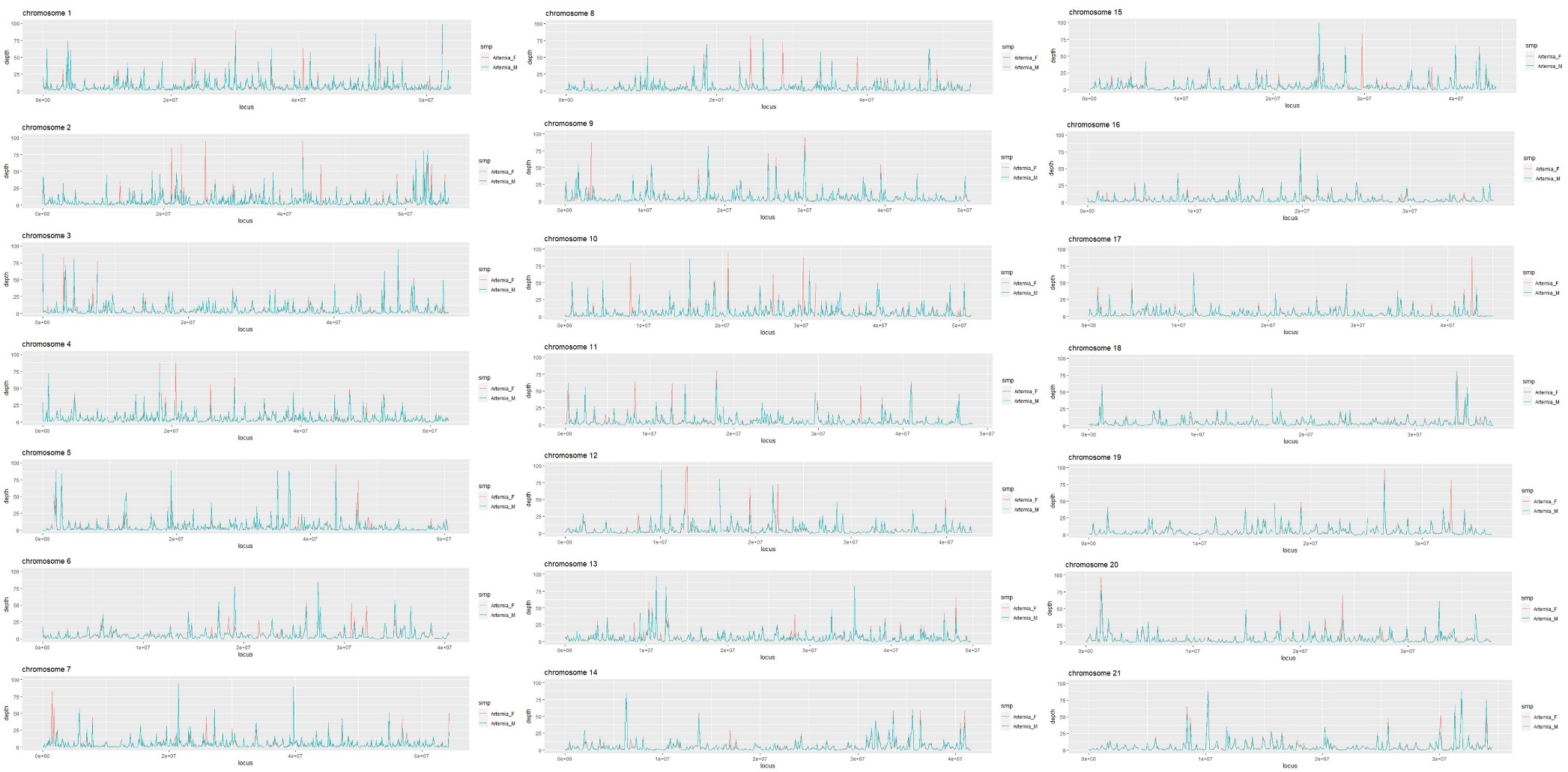


**Figure S6.** Coverage depths of female and male whole transcriptome sequencing (RNA-Seq) data to final 21 chromosomes of *Artemia franciscana*. Scarlet color plots represent female mapping reads, and mint color plots represent male mapping reads.


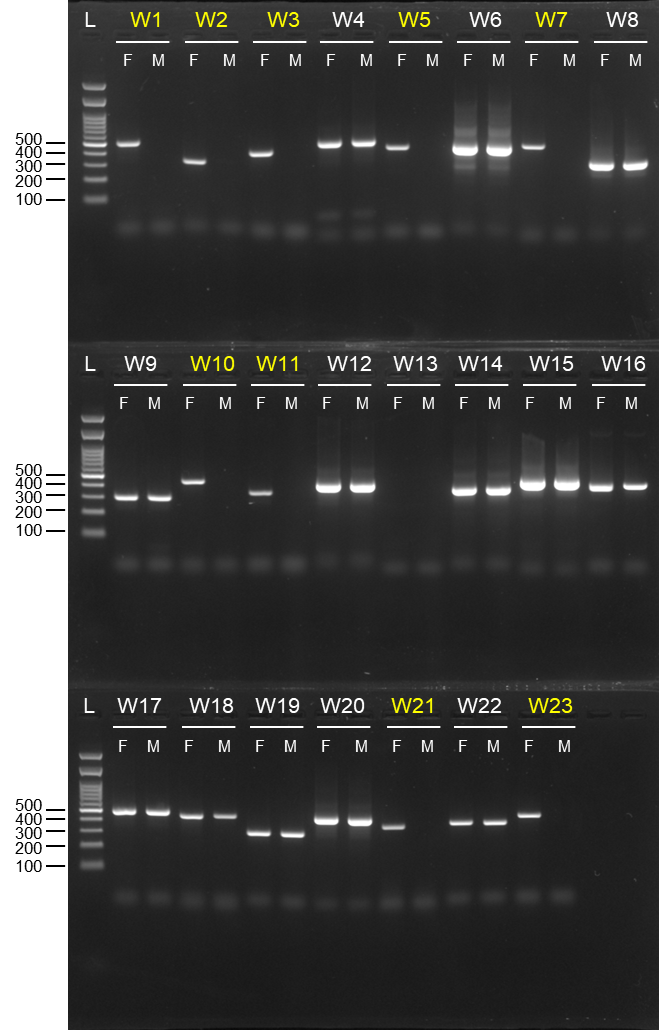


**Figure S7.** PCR validation of W chromosome-specific primers for female (F) and male (M) *Artemia franciscana*. Electrophoresis was conducted on 2% agarose gels with 100 bp+3K DNA ladder (L). The primers that distinguish between female and male are marked in yellow.


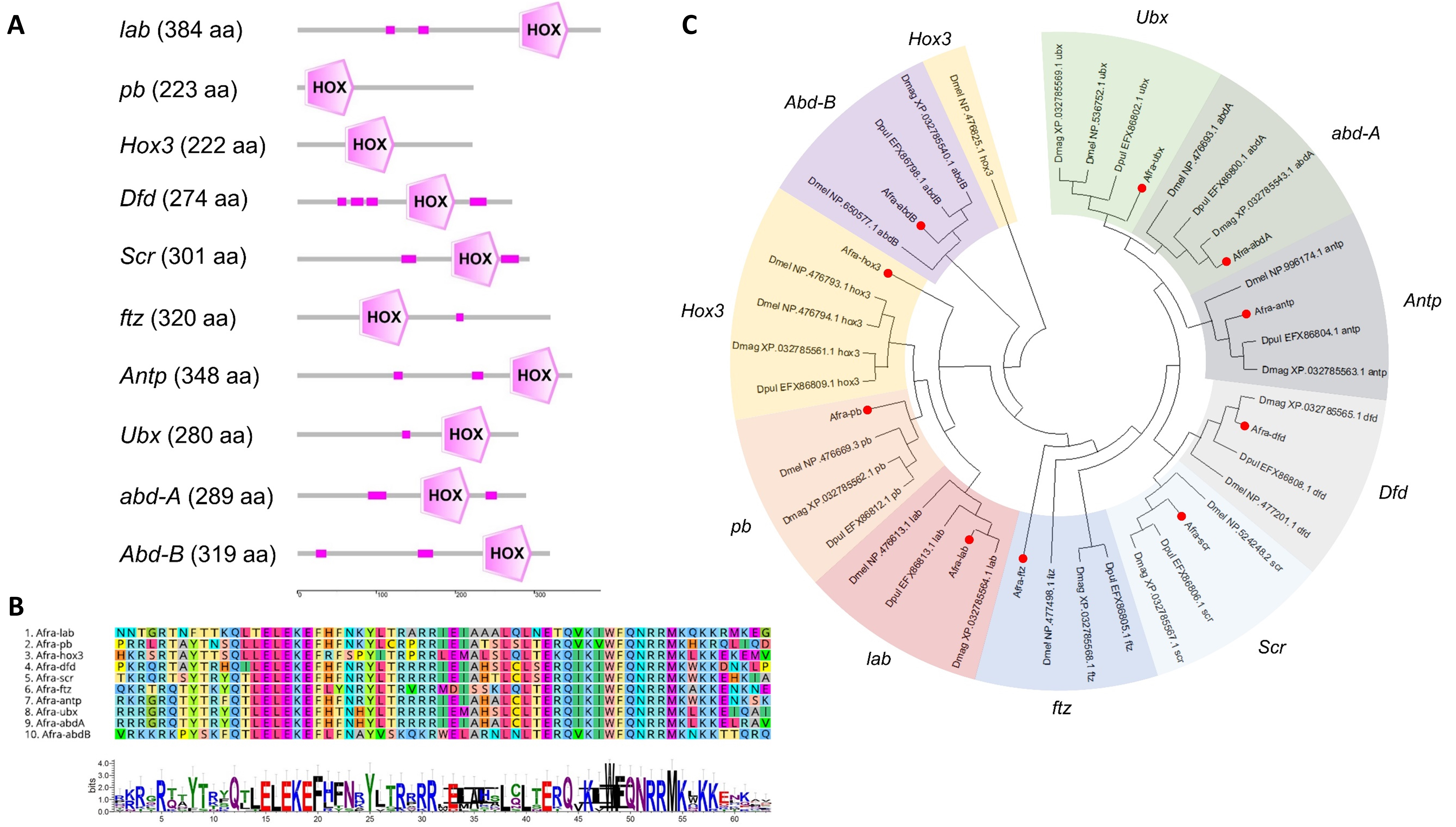


**Figure S8.** Homeodomain in *Artemia franciscana****.* (A)** Domain architecture of ten homeobox (*Hox*) genes identified in *A. franciscana.* **(B)** Multiple sequence alignment of homeodomain amino acid sequences from *A. franciscana.* **(C)** Phylogenetic tree of homeodomain of *A. franciscana* and three other arthropods. Red circles indicate the positions of *A. franciscana Hox* genes*.*


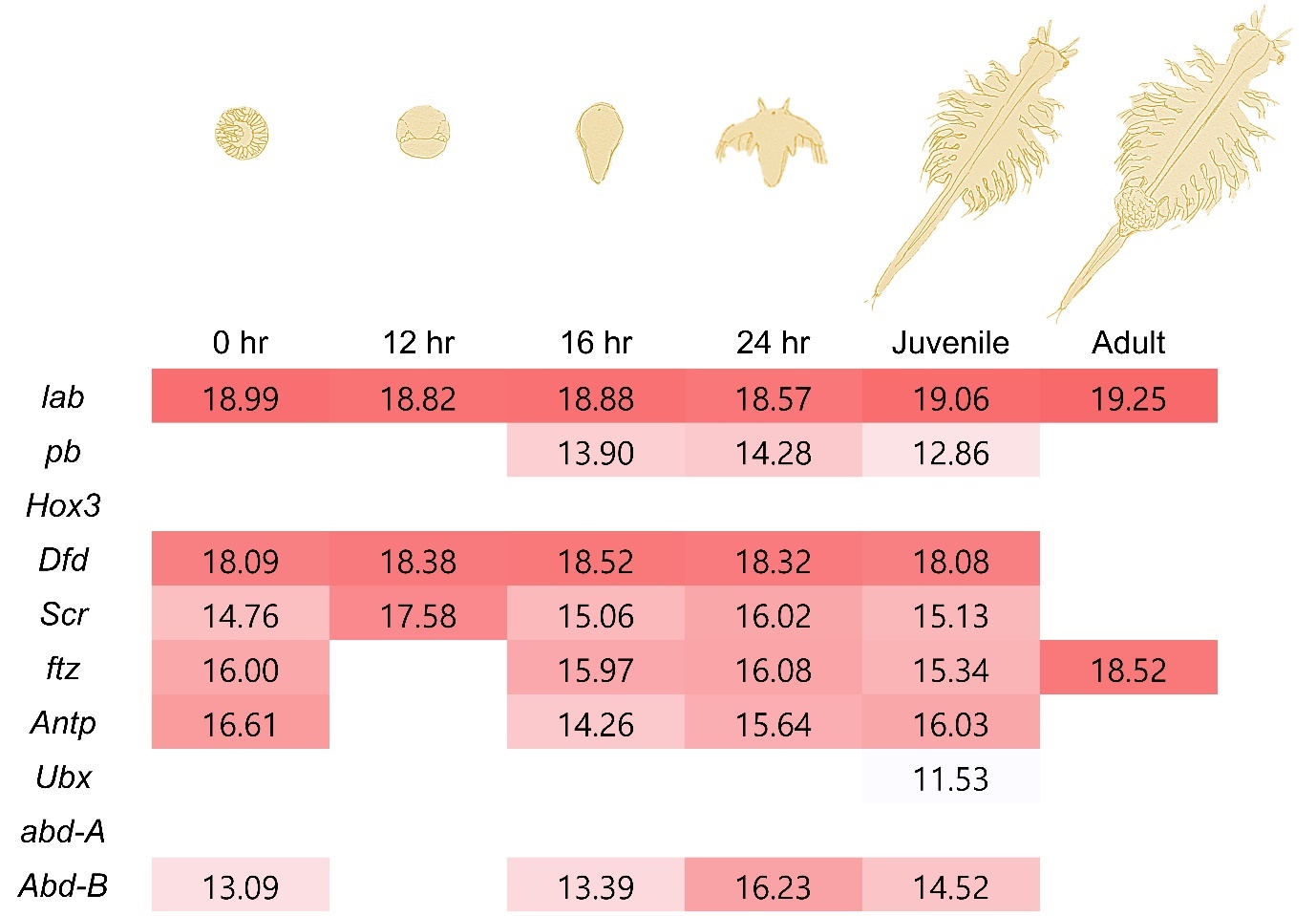


**Figure S9.** The RNA-Seq expression profiles of homeobox (*Hox*) genes in six developmental stages of *Artemia franciscana*. The color intensities and numbers represent log2 transcripts per million (TPM) values.

**
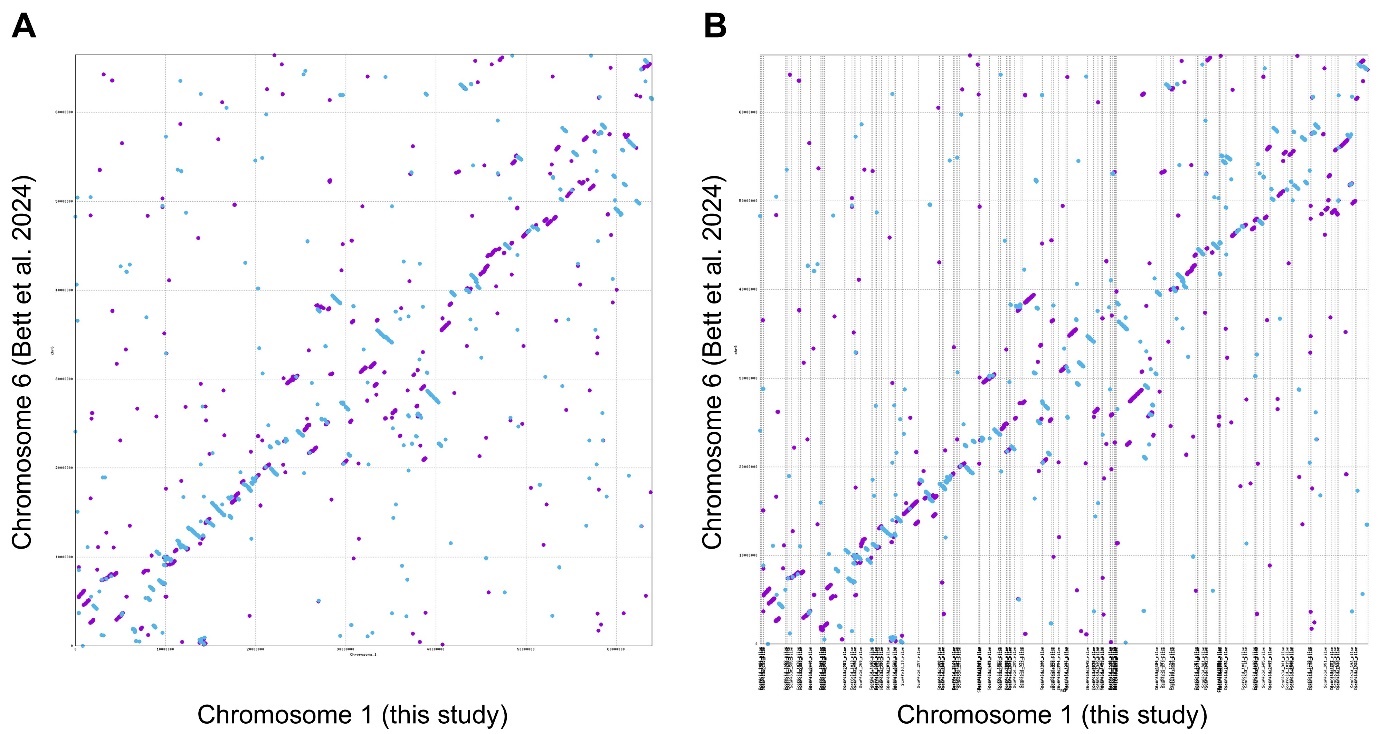
**

**Figure S10.** Mummer plots for comparison of Z chromosomes from two *Artemia franciscana* genome assemblies. **(A)** RagTag scaffold and **(B)** contig-level chromosome 1 from this study aligned against chromosome 6 from Bett et al. 2024 [S1].

**Table S1.** Sequencing data generated for *Artemia franciscana* genome assembly and annotation.

| **Library type** | **Platform** | **Number of samples** | **Number of cells** | **Number of reads** | **Number of bases** | **Purpose** | **Data source** |
| --- | --- | --- | --- | --- | --- | --- | --- |
| Short reads | Illumina HiSeq | 5 females | – | 475,508,298 | 22,814,108,862 | Polishing | Jo et al. [S2] |
| Long reads | PacBio Sequel | 14 females | 2 | 6,065,749 | 599,090,613,884 | Genome assembly | This study |
| 10x linked-reads | Illumina NovaSeq | 1 female | – | 414,237,254 | 62,549,825,354 | Genome scaffolding | This study |
| Hi-C | Illumina NovaSeq | 66 females | – | 686,076,522 | 103,597,554,822 | Hi-C map generation | This study |
| Iso-seq | PacBio Sequel | 4 of each sex | 2 | 678,253 | 2,128,734,891 | Annotation | Jo et al. [S3] |
| RNA-seq | Illumina NovaSeq | 4 of each sex | – | 227,251,536 | 22,952,405,136 | Annotation | Jo et al. [S3] |
| RNA-seq | Illumina HiSeq | 1 of each develop-mental stage | – | 116,801,504 | 23,555,443,376 | Annotation | This study |

**Table S2.** Summary statistics of intermediate assemblies of *Artemia franciscana* genome.

|  | **Peregrine+PurgeHaplotigs** | **+ARCS-Tigmint** | **+Pilon** | **+RagTag** |
| --- | --- | --- | --- | --- |
| Number of scaffolds | 9,312 | 10,751 | 10,665 | 8,033 |
| Total size of scaffolds (bp) | 1,276,739,447 | 1,277,043,447 | 1,272,440,009 | 1,272,703,209 |
| Longest scaffold (bp) | 2,888,585 | 5,911,916 | 5,896,170 | 71,465,782 |
| Number of scaffolds > 1 Mb | 119 | 370 | 370 | 45 |
| Number of scaffolds > 10 Mb | 0 | 0 | 0 | 21 |
| N50 scaffold length (bp) | 406,713 | 1,116,719 | 1,105,716 | 48,312,810 |
| L50 scaffold count | 911 | 325 | 325 | 11 |
| Scaffold GC content (%) | 34.73 | 34.72 | 34.74 | 34.74 |

**Table S3.** Lengths of 21 chromosome-level scaffolds (over 10 Mb) of *Artemia franciscana* genome.

| **No.** | **Scaffold name** | **Length (bp)** |
| --- | --- | --- |
| 1 | Chromosome_1 | 63,986,657 |
| 2 | Chromosome_2 | 67,337,973 |
| 3 | Chromosome_7 | 64,441,807 |
| 4 | Chromosome_4 | 63,039,847 |
| 5 | Chromosome_5 | 60,874,063 |
| 6 | Chromosome_3 | 55,982,255 |
| 7 | Chromosome_8 | 54,142,518 |
| 8 | Chromosome_10 | 51,680,706 |
| 9 | Chromosome_9 | 50,985,676 |
| 10 | Chromosome_13 | 49,921,436 |
| 11 | Chromosome_11 | 48,312,810 |
| 12 | Chromosome_17 | 45,252,357 |
| 13 | Chromosome_15 | 44,466,459 |
| 14 | Chromosome_12 | 42,830,064 |
| 15 | Chromosome_14 | 41,777,601 |
| 16 | Chromosome_6 | 40,553,139 |
| 17 | Chromosome_20 | 37,933,217 |
| 18 | Chromosome_16 | 37,925,480 |
| 19 | Chromosome_18 | 37,334,939 |
| 20 | Chromosome_19 | 36,455,688 |
| 21 | Chromosome_21 | 34,686,959 |
| Total |  | 1,029,921,651 |

**Table S4.** Benchmarking Universal Single-Copy Orthologs (BUSCO) completeness of the *Artemia franciscana* genome and transcriptome.

| **Mode** | **Genome** | | **Transcriptome** | |
| --- | --- | --- | --- | --- |
| **(Eukaryota_odb10)** | **Number** | **Percentage (%)** | **Number** | **Percentage (%)** |
| Complete BUSCOs (C) | 232 | 91.0 | 209 | 82.0 |
| Complete and single-copy BUSCOs (S) | 217 | 85.1 | 127 | 49.8 |
| Complete and duplicated BUSCOs (D) | 15 | 5.9 | 82 | 32.2 |
| Fragmented BUSCOs (F) | 14 | 5.5 | 19 | 7.5 |
| Missing BUSCOs (M) | 9 | 3.5 | 27 | 10.5 |
| Total BUSCO groups searched | 255 | – | 255 | – |

**Table S5.** Assessment for genome assembly of *Artemia franciscana* using Merqury.

| **Quality Value (QV)** | **k-mer error rate** | **k-mer completeness (%)** |
| --- | --- | --- |
| 47.624 | 1.72821e-05 | 68.808 |

**Table S6.** Statistics of repetitive elements in *Artemia franciscana* genome.

| **Class** |  | **Number of elements** | **Masked length (bp)** | **Percentage (%)** |
| --- | --- | --- | --- | --- |
| DNA elements |  | 305,818 | 91,098,092 | 7.16% |
|  | CMC-EnSpm | 89,986 | 23,050,261 | 1.81% |
|  | hAT-Tip100 | 59,338 | 11,522,126 | 0.91% |
|  | Maverick | 14,451 | 8,585,144 | 0.68% |
| LINEs |  | 349,168 | 162,126,824 | 12.75% |
|  | RTE-BovB | 107,590 | 45,988,051 | 3.62% |
|  | L2 | 58,154 | 25,760,406 | 2.03% |
|  | Penelope | 41,175 | 21,251,115 | 1.67% |
| LTR elements |  | 18,607 | 10,107,428 | 0.80% |
|  | Gypsy | 10,855 | 7,234,534 | 0.57% |
|  | Pao | 2,302 | 836,330 | 0.07% |
| RC | Helitron | 209,282 | 112,046,486 | 8.81% |
| SINEs |  | 15,236 | 3,169,347 | 0.0025 |
|  | 5S | 404 | 201,414 | 0.02% |
|  | tRNA | 14,832 | 2,967,933 | 0.23% |
| Unknown |  | 1,524,363 | 444,582,868 | 34.96% |
| Total interspersed repeats | | 2,422,474 | 823,131,045 | 64.73% |
| Low complexity |  | 35,370 | 1,945,655 | 0.15% |
| Satellites |  | 20,357 | 3,566,533 | 0.28% |
| Simple repeats |  | 253,153 | 20,984,228 | 1.65% |
| rRNAs |  | 11,500 | 2,034,555 | 0.16% |
| snRNAs |  | 2,519 | 526,555 | 0.04% |
| tRNAs |  | 12,269 | 3,075,410 | 0.24% |
| Total |  | 2,757,642 | 855,263,981 | 67.26% |

**Table S7.** Information of 14 crustacean species used for orthologous analysis.

| **Class** | **Order** | **Species** | **Assembly** |
| --- | --- | --- | --- |
| Branchiopoda | Anostraca | *Artemia franciscana* | This study |
|  | Cladocera | *Daphnia pulex* | GCA_021134715.1 (NCBI) |
|  |  | *Daphnia magna* | GCA_020631705.2 (Ensembl) |
|  | Spinicaudata | *Eulimnadia texana* | Baldwin-Brown et al. [S4] |
|  | Notostraca | *Lepidurus arcticus* | Savojardo et al. [S5] |
|  |  | *Lepidurus apus lubbocki* | Savojardo et al. [S5] |
| Malacostraca | Decapoda | *Procambarus clarkii* | GCA_020424385.2 (NCBI) |
|  |  | *Eriocheir sinensis* | GCA_024679095.1 (NCBI) |
|  |  | *Penaeus vannamei* | GCA_003789085.1 (Ensembl) |
|  | Amphipoda | *Hyalella azteca* | GCA_000764305.4 (Ensembl) |
|  |  | *Trinorchestia longiramus* | GCA_006783055.1 (Ensembl) |
| Hexanauplia-Copepoda | Harpacticoida | *Tigriopus californicus* | GCA_007210705.1 (Ensembl) |
|  | Calanoida | *Eurytemora affinis* | GCA_000591075.2 (Ensembl) |
| Ostracoda | Podocopida | *Cyprideis torosa* | GCA_905338395.1 (NCBI) |

**Table S8.** Summary of gene families of *Artemia franciscana* and other crustacean species.

|  | **Number of genes** | **Number of genes in orthogroups** | **Number of unassigned genes** | **Percentage of genes in orthogroups** | **Percentage of unassigned genes** | **Number of orthogroups containing species** | **Percentage of orthogroups containing species** | **Number of species-specific orthogroups** | **Number of genes in species-specific orthogroups** | **Percentage of genes in species-specific orthogroups** |
| --- | --- | --- | --- | --- | --- | --- | --- | --- | --- | --- |
| *Artemia franciscana* | 27,849 | 25,808 | 2,041 | 92.7 | 7.3 | 8,223 | 31.4 | 1,300 | 7,373 | 26.5 |
| *Daphnia magna* | 16,891 | 16,376 | 515 | 97 | 3 | 9,491 | 36.3 | 380 | 1,914 | 11.3 |
| *Daphnia pulex* | 27,985 | 27,287 | 698 | 97.5 | 2.5 | 9,668 | 37 | 683 | 3,208 | 11.5 |
| *Eulimnadia texana* | 23,965 | 21,025 | 2,940 | 87.7 | 12.3 | 8,816 | 33.7 | 772 | 3,168 | 13.2 |
| *Lepidurus apus lubbocki* | 16,289 | 14,385 | 1,904 | 88.3 | 11.7 | 9,245 | 35.4 | 315 | 973 | 6 |
| *Lepidurus arcticus* | 10,669 | 10,135 | 534 | 95 | 5 | 7,802 | 29.8 | 62 | 182 | 1.7 |
| *Eriocheir sinensis* | 54,884 | 52,196 | 2,688 | 95.1 | 4.9 | 11,246 | 43 | 1,108 | 12,507 | 22.8 |
| *Procambarus clarkii* | 26,417 | 17,966 | 8,451 | 68 | 32 | 10,666 | 40.8 | 329 | 1,587 | 6 |
| *Penaeus vannamei* | 24,987 | 21,369 | 3,618 | 85.5 | 14.5 | 10,845 | 41.5 | 496 | 2,094 | 8.4 |
| *Hyalella azteca* | 18,608 | 16,670 | 1,938 | 89.6 | 10.4 | 10,804 | 41.3 | 233 | 1,015 | 5.5 |
| *Trinorchestia longiramus* | 26,080 | 21,029 | 5,051 | 80.6 | 19.4 | 10,107 | 38.6 | 263 | 2,386 | 9.1 |
| *Eurytemora affinis* | 20,716 | 16,122 | 4,594 | 77.8 | 22.2 | 9,114 | 34.9 | 651 | 2,666 | 12.9 |
| *Tigriopus californicus* | 15,577 | 13,251 | 2,326 | 85.1 | 14.9 | 8,372 | 32 | 350 | 1,426 | 9.2 |
| *Cyprideis torosa* | 17,868 | 14,590 | 3,278 | 81.7 | 18.3 | 7,487 | 28.6 | 737 | 2,892 | 16.2 |

**Table S9.** Enriched Gene Ontology (GO) terms of expanded gene families in the *Artemia franciscana* genome among 14 crustacean species.

| **GO category** | **GO ID** | **GO name** | **P-value** | **Number of genes** |
| --- | --- | --- | --- | --- |
| Biological process | GO:0007526 | larval somatic muscle development | 1.11E-19 | 26 |
|  | GO:0000281 | mitotic cytokinesis | 1.45E-19 | 24 |
|  | GO:0006334 | nucleosome assembly | 4.83E-16 | 27 |
|  | GO:0006096 | glycolytic process | 7.09E-12 | 25 |
|  | GO:0006352 | DNA-templated transcription initiation | 3.38E-11 | 29 |
|  | GO:0043063 | intercellular bridge organization | 3.48E-10 | 8 |
|  | GO:0008608 | attachment of spindle microtubules to kinetochore | 3.32E-07 | 8 |
|  | GO:0007111 | meiosis II cytokinesis | 5.35E-07 | 6 |
|  | GO:0007140 | male meiotic nuclear division | 7.30E-07 | 8 |
|  | GO:0007094 | mitotic spindle assembly checkpoint signaling | 2.78E-06 | 8 |
|  | GO:0015074 | DNA integration | 6.04E-06 | 19 |
|  | GO:0009247 | glycolipid biosynthetic process | 1.43E-05 | 14 |
|  | GO:0030866 | cortical actin cytoskeleton organization | 3.65E-04 | 6 |
|  | GO:0098974 | postsynaptic actin cytoskeleton organization | 4.34E-03 | 2 |
|  | GO:1905612 | positive regulation of mRNA cap binding | 4.34E-03 | 2 |
|  | GO:0009792 | embryo development ending in birth or egg hatching | 1.04E-02 | 6 |
|  | GO:0000723 | telomere maintenance | 1.17E-02 | 9 |
|  | GO:0060964 | regulation of miRNA-mediated gene silencing | 1.24E-02 | 2 |
|  | GO:0002191 | cap-dependent translational initiation | 1.24E-02 | 2 |
|  | GO:0043547 | positive regulation of GTPase activity | 3.41E-02 | 5 |
|  | GO:2001256 | regulation of store-operated calcium entry | 2.38E-02 | 2 |
|  | GO:0040011 | locomotion | 2.87E-02 | 6 |
|  | GO:1900087 | positive regulation of G1/S transition of mitotic cell cycle | 2.38E-02 | 2 |
|  | GO:0032508 | DNA duplex unwinding | 1.93E-02 | 6 |
| Molecular function | GO:0004619 | phosphoglycerate mutase activity | 6.44E-27 | 25 |
|  | GO:0046982 | protein heterodimerization activity | 3.63E-22 | 54 |
|  | GO:0004082 | bisphosphoglycerate mutase activity | 8.72E-11 | 10 |
|  | GO:0001733 | galactosylceramide sulfotransferase activity | 3.49E-09 | 13 |
|  | GO:0030527 | structural constituent of chromatin | 3.62E-09 | 26 |
|  | GO:0098973 | structural constituent of postsynaptic actin cytoskeleton | 4.34E-03 | 2 |
|  | GO:0004190 | aspartic-type endopeptidase activity | 7.22E-03 | 6 |
|  | GO:0035256 | G protein-coupled glutamate receptor binding | 2.38E-02 | 2 |
|  | GO:0031369 | translation initiation factor binding | 4.01E-02 | 3 |
|  | GO:0016779 | nucleotidyltransferase activity | 4.51E-02 | 15 |
| Cellular component | GO:0005669 | transcription factor TFIID complex | 9.62E-24 | 29 |
|  | GO:0005704 | polytene chromosome band | 2.18E-20 | 26 |
|  | GO:0097433 | dense body | 1.78E-18 | 15 |
|  | GO:0005925 | focal adhesion | 2.01E-14 | 15 |
|  | GO:0000786 | nucleosome | 9.22E-09 | 27 |
|  | GO:0005884 | actin filament | 5.32E-04 | 6 |
|  | GO:0016281 | eukaryotic translation initiation factor 4F complex | 2.38E-02 | 2 |

**Table S10.** Contig information and W contigs identification within RagTag scaffolded chromosome 1 based on the AGP format file.

| **Start** | **End** | **Contig ID** | **Size** | **Orientation** | **Comment** |
| --- | --- | --- | --- | --- | --- |
| 1 | 49738 | Scaffold_2024_pilon | 49738 | + |  |
| 49839 | 143249 | Scaffold_1436_pilon | 93411 | - |  |
| 143350 | 216768 | Scaffold_1599_pilon | 73419 | + |  |
| 216869 | 332072 | Scaffold_1742_pilon | 115204 | - |  |
| 332173 | 377169 | Scaffold_3753_pilon | 44997 | - |  |
| 377270 | 2666179 | Scaffold_182_pilon | 2288910 | + |  |
| 2666280 | 2813101 | Scaffold_1152_pilon | 146822 | - |  |
| 2813202 | 2875117 | Scaffold_1768_pilon | 61916 | + |  |
| 2875218 | 3031527 | Scaffold_1120_pilon | 156310 | + |  |
| 3031628 | 3281909 | Scaffold_1005_pilon | 250282 | + |  |
| 3282010 | 3999440 | Scaffold_535_pilon | 717431 | + |  |
| 3999541 | 4218793 | Scaffold_1073_pilon | 219253 | + |  |
| 4218894 | 4259925 | Scaffold_2282_pilon | 41032 | - |  |
| 4260026 | 4305610 | Scaffold_2130_pilon | 45585 | + |  |
| 4305711 | 5281453 | Scaffold_262_pilon | 975743 | + |  |
| 5281554 | 5319932 | Scaffold_2418_pilon | 38379 | + |  |
| 5320033 | 5323437 | Scaffold_8543_pilon | 3405 | - |  |
| 5323538 | 6363125 | Scaffold_649_pilon | 1039588 | + |  |
| 6363226 | 6421170 | Scaffold_1846_pilon | 57945 | - |  |
| 6421271 | 6574305 | Scaffold_1391_pilon | 153035 | - |  |
| 6574406 | 6610673 | Scaffold_2567_pilon | 36268 | - |  |
| 6610774 | 6699296 | Scaffold_1474_pilon | 88523 | + |  |
| 6699397 | 6787314 | Scaffold_1471_pilon | 87918 | - |  |
| 6787415 | 6802809 | Scaffold_6961_pilon | 15395 | - |  |
| 6802910 | 8910031 | Scaffold_205_pilon | 2107122 | - |  |
| 8910132 | 8937903 | Scaffold_3553_pilon | 27772 | - |  |
| 8938004 | 9688075 | Scaffold_260_pilon | 750072 | + |  |
| 9688176 | 9943971 | Scaffold_1010_pilon | 255796 | + |  |
| 9944072 | 9991392 | Scaffold_2079_pilon | 47321 | + |  |
| 9991493 | 10607587 | Scaffold_496_pilon | 616095 | + |  |
| 10607688 | 11713403 | Scaffold_369_pilon | 1105716 | - |  |
| 11713504 | 11863013 | Scaffold_1142_pilon | 149510 | - |  |
| 11863114 | 12167592 | Scaffold_808_pilon | 304479 | + |  |
| 12167693 | 12236538 | Scaffold_1653_pilon | 68846 | - |  |
| 12236639 | 12265218 | Scaffold_3356_pilon | 28580 | + |  |
| 12265319 | 12514323 | Scaffold_1050_pilon | 249005 | + |  |
| 12514424 | 12768750 | Scaffold_834_pilon | 254327 | - |  |
| 12768851 | 12807586 | Scaffold_2398_pilon | 38736 | + |  |
| 12807687 | 13467403 | Scaffold_280_pilon | 659717 | + |  |
| 13467504 | 13482377 | Scaffold_7071_pilon | 14874 | - |  |
| 13482478 | 13494029 | Scaffold_7513_pilon | 11552 | + |  |
| 13494130 | 14125743 | Scaffold_872_pilon | 631614 | + |  |
| 14125844 | 14207634 | Scaffold_1532_pilon | 81791 | + |  |
| 14207735 | 14249344 | Scaffold_2260_pilon | 41610 | - |  |
| 14249445 | 14268426 | Scaffold_5942_pilon | 18982 | + |  |
| 14268527 | 14978488 | Scaffold_236_pilon | 709962 | - |  |
| 14978589 | 16682941 | Scaffold_10_pilon | 1704353 | - |  |
| 16683042 | 18836020 | Scaffold_257_pilon | 2152979 | - |  |
| 18836121 | 18881875 | Scaffold_2122_pilon | 45755 | - |  |
| 18881976 | 19191754 | Scaffold_966_pilon | 309779 | + |  |
| 19191855 | 19215276 | Scaffold_4504_pilon | 23422 | - |  |
| 19215377 | 19273342 | Scaffold_1848_pilon | 57966 | - |  |
| 19273443 | 20370218 | Scaffold_561_pilon | 1096776 | + |  |
| 20370319 | 20414571 | Scaffold_2171_pilon | 44253 | - |  |
| 20414672 | 20454977 | Scaffold_2319_pilon | 40306 | - |  |
| 20455078 | 20692858 | Scaffold_1160_pilon | 237781 | - |  |
| 20692959 | 20775321 | Scaffold_1524_pilon | 82363 | + |  |
| 20775422 | 20973245 | Scaffold_992_pilon | 197824 | + |  |
| 20973346 | 23018002 | Scaffold_291_pilon | 2044657 | + |  |
| 23018103 | 23058602 | Scaffold_2309_pilon | 40500 | + |  |
| 23058703 | 23058966 | Scaffold_10320_pilon | 264 | + |  |
| 23059067 | 23092666 | Scaffold_2782_pilon | 33600 | + |  |
| 23092767 | 23164630 | Scaffold_1625_pilon | 71864 | + |  |
| 23164731 | 24101896 | Scaffold_222_pilon | 937166 | + |  |
| 24101997 | 24132087 | Scaffold_3174_pilon | 30091 | - |  |
| 24132188 | 24160443 | Scaffold_3467_pilon | 28256 | - |  |
| 24160544 | 25097123 | Scaffold_665_pilon | 936580 | + |  |
| 25097224 | 25262504 | Scaffold_1083_pilon | 165281 | + |  |
| 25262605 | 25325037 | Scaffold_2688_pilon | 62433 | + |  |
| 25325138 | 25926756 | Scaffold_566_pilon | 601619 | + |  |
| 25926857 | 25935508 | Scaffold_7638_pilon | 8652 | + |  |
| 25935609 | 25945269 | Scaffold_7716_pilon | 9661 | + |  |
| 25945370 | 25980142 | Scaffold_2689_pilon | 34773 | + |  |
| 25980243 | 26224134 | Scaffold_928_pilon | 243892 | + |  |
| 26224235 | 26291353 | Scaffold_1691_pilon | 67119 | + |  |
| 26291454 | 26356038 | Scaffold_1730_pilon | 64585 | - |  |
| 26356139 | 26787194 | Scaffold_973_pilon | 431056 | - |  |
| 26787295 | 27429177 | Scaffold_285_pilon | 641883 | - |  |
| 27429278 | 27631196 | Scaffold_972_pilon | 201919 | + |  |
| 27631297 | 29658316 | Scaffold_123_pilon | 2027020 | - |  |
| 29658417 | 29711839 | Scaffold_1938_pilon | 53423 | + |  |
| 29711940 | 30667208 | Scaffold_238_pilon | 955269 | + |  |
| 30667309 | 30865004 | Scaffold_989_pilon | 197696 | + |  |
| 30865105 | 30889825 | Scaffold_4221_pilon | 24721 | - |  |
| 30889926 | 31396251 | Scaffold_828_pilon | 506326 | + |  |
| 31396352 | 31423083 | Scaffold_3771_pilon | 26732 | + |  |
| 31423184 | 32167340 | Scaffold_484_pilon | 744157 | + |  |
| 32167441 | 32281569 | Scaffold_1293_pilon | 114129 | - |  |
| 32281670 | 32312801 | Scaffold_3044_pilon | 31132 | - |  |
| 32312902 | 34439590 | Scaffold_227_pilon | 2126689 | - |  |
| 34439691 | 34454151 | Scaffold_7142_pilon | 14461 | + |  |
| 34454252 | 35077878 | Scaffold_645_pilon | 623627 | + |  |
| 35077979 | 35102669 | Scaffold_4230_pilon | 24691 | + |  |
| 35102770 | 35133607 | Scaffold_3076_pilon | 30838 | + |  |
| 35133708 | 35452224 | Scaffold_723_pilon | 318517 | + |  |
| 35452325 | 35554770 | Scaffold_1373_pilon | 102446 | + |  |
| 35554871 | 35927812 | Scaffold_902_pilon | 372942 | + |  |
| 35927913 | 36028488 | Scaffold_1387_pilon | 100576 | - |  |
| 36028589 | 36052457 | Scaffold_4452_pilon | 23869 | - |  |
| 36052558 | 36834431 | Scaffold_485_pilon | 781874 | - |  |
| 36834532 | 36858532 | Scaffold_4408_pilon | 24001 | - |  |
| 36858633 | 36901957 | Scaffold_2192_pilon | 43325 | + |  |
| 36902058 | 37062110 | Scaffold_1278_pilon | 160053 | - |  |
| 37062211 | 37242264 | Scaffold_1439_pilon | 180054 | - |  |
| 37242365 | 37330961 | Scaffold_1473_pilon | 88597 | - |  |
| 37331062 | 37371514 | Scaffold_2311_pilon | 40453 | - |  |
| 37371615 | 37398798 | Scaffold_3667_pilon | 27184 | + |  |
| 37398899 | 37415313 | Scaffold_6719_pilon | 16415 | + |  |
| 37415414 | 37447948 | Scaffold_2885_pilon | 32535 | - |  |
| 37448049 | 37480243 | Scaffold_2922_pilon | 32195 | - |  |
| 37480344 | 41745404 | Scaffold_5_pilon | 4265061 | - |  |
| 41745505 | 41761133 | Scaffold_6859_pilon | 15629 | - |  |
| 41761234 | 42246981 | Scaffold_824_pilon | 485748 | + |  |
| 42247082 | 42494798 | Scaffold_849_pilon | 247717 | - |  |
| 42494899 | 43186819 | Scaffold_570_pilon | 691921 | + |  |
| 43186920 | 43469291 | Scaffold_1154_pilon | 282372 | - |  |
| 43469392 | 43501956 | Scaffold_2880_pilon | 32565 | - |  |
| 43502057 | 45879528 | Scaffold_23_pilon | 2377472 | + |  |
| 45879629 | 46024414 | Scaffold_1251_pilon | 144786 | - |  |
| 46024515 | 46069499 | Scaffold_2151_pilon | 44985 | + |  |
| 46069600 | 46102239 | Scaffold_2870_pilon | 32640 | + |  |
| 46102340 | 46652489 | Scaffold_410_pilon | 550150 | - |  |
| 46652590 | 46888198 | Scaffold_881_pilon | 235609 | + |  |
| 46888299 | 46983646 | Scaffold_1626_pilon | 95348 | - |  |
| 46983747 | 48257025 | Scaffold_392_pilon | 1273279 | + |  |
| 48257126 | 48261104 | Scaffold_8440_pilon | 3979 | - |  |
| 48261205 | 48290819 | Scaffold_3234_pilon | 29615 | - |  |
| 48290920 | 48291325 | Scaffold_10094_pilon | 406 | - |  |
| 48291426 | 48311145 | Scaffold_5750_pilon | 19720 | - |  |
| 48311246 | 48733908 | Scaffold_618_pilon | 422663 | + | W contig 1 |
| 48734009 | 48734384 | Scaffold_10142_pilon | 376 | + |  |
| 48734485 | 48800988 | Scaffold_1698_pilon | 66504 | - |  |
| 48801089 | 48877392 | Scaffold_1570_pilon | 76304 | - |  |
| 48877493 | 48893930 | Scaffold_6704_pilon | 16438 | - |  |
| 48894031 | 48909287 | Scaffold_6987_pilon | 15257 | - |  |
| 48909388 | 52472849 | Scaffold_27_pilon | 3563462 | + | W contig 2 |
| 52472950 | 52481478 | Scaffold_7841_pilon | 8529 | - |  |
| 52481579 | 52975667 | Scaffold_455_pilon | 494089 | - |  |
| 52975768 | 53053167 | Scaffold_1972_pilon | 77400 | - |  |
| 53053268 | 54447237 | Scaffold_581_pilon | 1393970 | - | W contig 3 |
| 54447338 | 56240529 | Scaffold_408_pilon | 1793192 | + |  |
| 56240630 | 58138516 | Scaffold_215_pilon | 1897887 | + | W contig 4 |
| 58138617 | 58408527 | Scaffold_1001_pilon | 269911 | + |  |
| 58408628 | 59294731 | Scaffold_418_pilon | 886104 | - |  |
| 59294832 | 59446973 | Scaffold_1132_pilon | 152142 | + |  |
| 59447074 | 59447798 | Scaffold_9636_pilon | 725 | + |  |
| 59447899 | 59452014 | Scaffold_8412_pilon | 4116 | + |  |
| 59452115 | 59469797 | Scaffold_6411_pilon | 17683 | + |  |
| 59469898 | 59472403 | Scaffold_8714_pilon | 2506 | + |  |
| 59472504 | 59567338 | Scaffold_1428_pilon | 94835 | + |  |
| 59567439 | 60237068 | Scaffold_324_pilon | 669630 | - |  |
| 60237169 | 60919181 | Scaffold_685_pilon | 682013 | + |  |
| 60919282 | 60968188 | Scaffold_2047_pilon | 48907 | - |  |
| 60968289 | 62342260 | Scaffold_452_pilon | 1373972 | + |  |
| 62342361 | 62543003 | Scaffold_1108_pilon | 200643 | - | W contig 5 |
| 62543104 | 62984124 | Scaffold_901_pilon | 441021 | - |  |
| 62984225 | 63364697 | Scaffold_1019_pilon | 380473 | - |  |
| 63364798 | 63392028 | Scaffold_3511_pilon | 27231 | - |  |
| 63392129 | 63414612 | Scaffold_4867_pilon | 22484 | - |  |
| 63414713 | 63498252 | Scaffold_2039_pilon | 83540 | + |  |
| 63498353 | 65218695 | Scaffold_506_pilon | 1720343 | - |  |
| 65218796 | 65427392 | Scaffold_1139_pilon | 208597 | + |  |
| 65427493 | 65469969 | Scaffold_4788_pilon | 42477 | - |  |
| 65470070 | 66993778 | Scaffold_113_pilon | 1523709 | + |  |
| 66993879 | 67637600 | Scaffold_851_pilon | 643722 | - |  |
| 67637701 | 67639985 | Scaffold_8757_pilon | 2285 | + |  |
| 67640086 | 67964824 | Scaffold_871_pilon | 324739 | - |  |
| 67964925 | 68276005 | Scaffold_735_pilon | 311081 | - |  |
| 68276106 | 68335169 | Scaffold_1820_pilon | 59064 | - |  |
| 68335270 | 69759111 | Scaffold_318_pilon | 1423842 | - |  |
| 69759212 | 70146167 | Scaffold_693_pilon | 386956 | - |  |
| 70146268 | 70200747 | Scaffold_1922_pilon | 54480 | - |  |
| 70200848 | 71465782 | Scaffold_738_pilon | 1264935 | - |  |

**Table S11.** Candidate sex-determination genes with more than two-fold differences in expression between female and male *Artemia franciscana*.

| **SeqID** | **Description** | **Expression (WTS)** | **Chromosome (WGS)** |
| --- | --- | --- | --- |
| Artemia00003275 | actin, muscle | F | W2 |
| Artemia00003276 | sprT-like domain-containing protein Spartan | F | W2 |
| Artemia00003277 | 52 kDa repressor of the inhibitor of the protein kinase-like | F | W2 |
| Artemia00003293 | protein FAM200A-like | F | W2 |
| Artemia00003294 | protein amalgam-like | F | W2 |
| Artemia00003295 | RNA-directed DNA polymerase from mobile element jockey-like | F | W2 |
| Artemia00003296 | ero1-like protein | F | W2 |
| Artemia00003297 | hypothetical protein X777_15130 | F | W2 |
| Artemia00003331 | dual specificity tyrosine-phosphorylation-regulated kinase 2 | F | W3 |
| Artemia00003332 | Striatin-interacting protein 1 | F | W3 |
| Artemia00003333 | Striatin-interacting protein 1 | F | W3 |
| Artemia00003335 | Transposon Ty3-I Gag-Pol polyprotein | F | W3 |
| Artemia00003339 | Methylmalonyl-CoA mutase, mitochondrial | M | Z |
| Artemia00003340 | rhythmically expressed gene 5 protein | M | Z |
| Artemia00003372 | zinc finger MYM-type protein 1-like | F | W4 |
| Artemia00003373 | Retrovirus-related Pol polyprotein from transposon 297 | F | W4 |
| Artemia00003377 | uncharacterized protein LOC114158190 | F | W4 |
| Artemia00003378 | PREDICTED: uncharacterized protein LOC109462593 | F | W4 |
| Artemia00003382 | hypothetical protein | F | W4 |
| Artemia00003383 | protein canopy 4 | F | W4 |
| Artemia00003402 | LysR family transcriptional regulator | F | W4 |
| Artemia00003403 | Retrovirus-related Pol polyprotein from transposon 412 | F | W4 |
| Artemia00003404 | Retrovirus-related Pol polyprotein from transposon 17.6 | F | W4 |
| Artemia00003405 | 10 kDa heat shock protein, mitochondrial | F | W4 |
| Artemia00003406 | 60 kDa heat shock protein, mitochondrial | F | W4 |
| Artemia00003411 | Phosphatidate cytidylyltransferase, mitochondrial | M | Z |
| Artemia00003412 | Nucleotide-binding oligomerization domain-containing 1-like protein | M | Z |
| Artemia00003414 | Mitochondrial tRNA-specific 2-thiouridylase 1 | M | Z |
| Artemia00003586 | ---NA--- | F | Z |
| Artemia00003587 | aliphatic sulfonate ABC transporter substrate-binding protein | F | Z |
| Artemia00003599 | Chaoptin | M | Z |
| Artemia00003600 | chaoptin | M | Z |
| Artemia00003626 | dual specificity mitogen-activated protein kinase kinase 7-like | F | Z |
| Artemia00003647 | mannosyl-oligosaccharide 1,2-alpha-mannosidase IB | M | PAR2 |
| Artemia00003648 | DUF4129 domain-containing protein | M | PAR2 |

**Table S12.** PCR primers designed from genes probably located on the W chromosome of *Artemia franciscana*.

| **Target gene** | **Primer name** | **Forward sequence (5′ to 3′)** | **Reverse sequence (5′ to 3′)** | **Expected product size (bp)** |
| --- | --- | --- | --- | --- |
| actin, muscle | W1 | GGAGAAAGAGAGACTTAAGGGC | AGTTGATGCTTCCGTTCAGC | 500 |
| sprT-like domain-containing protein Spartan | W2 | TGTCCGTTTTTGAGGATCTCCA | AGCCTCCTCTCCTAACTCCA | 335 |
| 52 kDa repressor of the inhibitor of the protein kinase-like | W3 | GCAGCCAAAATGCCTGGTAG | TCAGCTAGGCCAGAGTCTGT | 422 |
| protein FAM200A-like | W4 | TCCAGCTCGGTTTCAGAACA | CCCCTGGCCACCTTATTGTT | 489 |
| protein amalgam-like | W5 | ACCTGTATGTGGTTGCCTCA | AGCCCAAAACAACGGAGGAA | 438 |
| RNA-directed DNA polymerase from mobile element jockey-like | W6 | CCTGTCTAATGCTGTGGGGG | AGACGATGCATTACACCTACGT | 455 |
| ero1-like protein | W7 | CTGCAAATCAGACCGCTAACG | TCTTGCCTTGCCTCCTTCTG | 467 |
| hypothetical protein X777_15130 | W8 | ATCTTCGGCAAGGCTTGTCA | ACAAGACGGCTCTCAACAGG | 330 |
| dual specificity tyrosine-phosphorylation-regulated kinase 2 | W9 | TCACGGTTTTATCGAGCACCA | TATGTTTTCCCCGCCGAGAG | 310 |
| Striatin-interacting protein 1 | W10 | TCCTTAGTCCTCGGCACAGT | AGAGCGAGAAGGAGTGGAGA | 450 |
| Striatin-interacting protein 1 | W11 | ACCATGGACTACGCTCTTGC | TGTCCGTCAGGCTACTCTGA | 352 |
| Transposon Ty3-I Gag-Pol polyprotein | W12 | GCTTCAAGTTCCACAAGCGG | TACATGCACGGGCGAAGTAG | 441 |
| zinc finger MYM-type protein 1-like | W13 | TCCTTCCGAGCTATAGACCGT | TGGAAGTCCTAGTCCCTGCT | 479 |
| Retrovirus-related Pol polyprotein from transposon 297 | W14 | TCTCGCGAGTATTCCCGTTG | ACGAGAGGCAACCCACAAAA | 393 |
| uncharacterized protein LOC114158190 | W15 | GGCAAGCTATCATGGGGTGA | ACGATTAGGTCCTGGAGGCT | 485 |
| PREDICTED: uncharacterized protein LOC109462593 | W16 | GTTACCCCATCATCAGCCCC | TGGGATACGCTCGTTAGTCG | 399 |
| hypothetical protein | W17 | TGGGACAGGATTTGGGCATC | TCCAGCAGGCTTAGGCTCTA | 496 |
| protein canopy 4 | W18 | TTCGCTGATGGTGGATGCTT | TTGCTTGTTTCTTACCGCGC | 449 |
| LysR family transcriptional regulator | W19 | GGTCGCTCTCCATGAGAAGG | TCGACAATCCCAGCAGTCAA | 312 |
| Retrovirus-related Pol polyprotein from transposon 412 | W20 | AGCCAAGTTCGCAGCAGTAT | TTACCTGTCAGCAAGGCCTG | 455 |
| Retrovirus-related Pol polyprotein from transposon 17.6 | W21 | CGCCGCTGACAGATCTTCTT | CCGGTGAAGCAGGTTCTGAT | 369 |
| 10 kDa heat shock protein, mitochondrial | W22 | CGTTGTTTCTTGCACCTGGG | CGTCGATGAAGGGTTACTGGT | 400 |
| 60 kDa heat shock protein, mitochondrial | W23 | AACGTGTGTGGTTTGACAGT | TTTCCACGAGGTCAAACCGT | 469 |

**Table S13.** Ten typical homeobox (*Hox*) genes identified in *Artemia franciscana* chromosome 14.

| **Gene name (symbol)** | **Intron number** | **Positions** | **Strand** | **Size (aa)** |
| --- | --- | --- | --- | --- |
| Labial (*lab*) | 2 | 34909505–34940254 | + | 384 |
| Proboscipedia (*pb*) | 1 | 34813015–34858099 | + | 223 |
| *Hox3*/Zerknult (*zen*) | 1 | 23093326–23097634 | + | 222 |
| Deformed (*Dfd*) | 1 | 22950281–22993320 | + | 274 |
| Sex combs reduced (*Scr*) | 1 | 22864404–22895126 | + | 301 |
| fushi tarazu (*ftz*) | 3 | 22816116–22844533 | - | 320 |
| Antennapedia (*Antp*) | 1 | 22753563–22773475 | + | 348 |
| Ultrabithorax (*Ubx*) | 1 | 22682869–22711984 | + | 280 |
| Abdominal-A (*Abd-A*) | 1 | 22538981–22562508 | + | 289 |
| Abdominal-B (*Abd-B*) | 1 | 22007211–22045898 | + | 319 |

**Table S14.** List of arthropod species used for homeobox (*Hox*) gene analysis.

| **Taxon** | **Species** | **Source (GenBank acc. no.)** | **Assembly** | **Reference** |
| --- | --- | --- | --- | --- |
| Anostraca | *Artemia franciscana* | NCBI (GCA_032884065.1) | ASM3288406v1 | This study |
|  | *Artemia sinica* | NCBI (GCA_027921565.1) | ASM2792156v1 | Elkrewi et al. [S6] |
|  | *Branchinecta lindahli* | NCBI (GCA_023053555.1) | BRLI_1.1 | Kieran Blair et al. [S7] |
|  | *Branchinecta lynchi* | NCBI (GCA_023053575.1) | BRLY_1.1 | Kieran Blair et al. [S8] |
| Anomopoda | *Daphnia magna* | NCBI (GCA_020631705.2) | ASM2063170v1.1 | Kim et al. [S9] |
|  | *Daphnia pulex* | NCBI (GCA_021134715.1) | ASM2113471v1 | – |
| Spinicaudata | *Eulimnadia texana* | NCBI (GCA_002872375.1) | clam_shrimp_assembly_v0.1 | Baldwin-Brown et al. [S4] |
| Notostraca | *Lepidurus arcticus* | NCBI (GCA_003724045.1) | ASM372404v1 | Savojardo et al. [S5] |
|  | *Lepidurus apus apus* | NCBI (GCA_022832285.1) | Lapu_2021 | Luchetti et al. [S10] |
|  | *Triops cancriformis* | NCBI (GCA_020615345.1) | EvoH_TcaCAE_1.0 | Orr [S11] |
|  | *Triops longicaudatus* | NCBI (GCA_022885665.1) | TLON_2021 | Luchetti et al. [S10] |
| Copepoda | *Paracylopina nana* | NCBI (KT345727.1) | – | Kim et al. [S12] |
| Hexapoda | *Drosophila melanogaster* | FlyBase FB2022_01 (GCA_000001215.4) | Release 6 plus ISO1 MT | Gramates et al. [S13] |

**Supplemental references**

1. Bett, V.K., Macon, A., Vicoso, B., and Elkrewi, M. (2024). Chromosome-Level Assembly of *Artemia franciscana* Sheds Light on Sex Chromosome Differentiation. Genome Biol. Evol. *16*, evae006.
2. Jo, E., Lee, S.J., Choi, E., Kim, J., Lee, S.G., Lee, J.H., Kim, J.-H., and Park, H. (2021). Whole genome survey and microsatellite motif identification of *Artemia franciscana*. Biosci. Rep. *41*.
3. Jo, E., Lee, S.J., Choi, E., Kim, J., Lee, J.H., and Park, H. (2021). Sex-biased gene expression and isoform profile of Brine Shrimp *Artemia franciscana* by transcriptome analysis. Animals *11*, 2630.
4. Baldwin-Brown, J.G., Weeks, S.C., and Long, A.D. (2018). A new standard for crustacean genomes: the highly contiguous, annotated genome assembly of the clam shrimp *Eulimnadia texana* reveals *HOX* gene order and identifies the sex chromosome. Genome Biol. Evol. *10*, 143-156.
5. Savojardo, C., Luchetti, A., Martelli, P.L., Casadio, R., and Mantovani, B. (2019). Draft genomes and genomic divergence of two *Lepidurus* tadpole shrimp species (Crustacea, Branchiopoda, Notostraca). Mol. Ecol. Resour. *19*, 235-244.
6. Elkrewi, M., Khauratovich, U., Toups, M.A., Bett, V.K., Mrnjavac, A., Macon, A., Fraisse, C., Sax, L., Huylmans, A.K., and Hontoria, F. (2022). ZW sex-chromosome evolution and contagious parthenogenesis in *Artemia* brine shrimp. Genetics *222*, iyac123.
7. Kieran Blair, S.R., Schreier, A., Escalona, M., Finger, A.J., Joslin, S.E., Sahasrabudhe, R., Marimuthu, M.P., Nguyen, O., Chumchim, N., and Morris, E.R. (2023). A chromosome-level reference genome for the Versatile Fairy Shrimp, *Branchinecta lindahli*. J. Hered. *114*, 74-80.
8. Kieran Blair, S.R., Schreier, A., Escalona, M., Finger, A.J., Joslin, S.E., Sahasrabudhe, R., Marimuthu, M.P., Nguyen, O., Chumchim, N., and Morris, E.R. (2023). A draft reference genome of the Vernal Pool Fairy Shrimp, *Branchinecta lynchi*. J. Hered. *114*, 81-87.
9. Kim, D.H., Lee, B.Y., Kim, H.S., Jeong, C.B., Hwang, D.S., Kim, I.C., and Lee, J.S. (2018). Identification and characterization of homeobox (*Hox*) genes and conservation of the single *Hox* cluster (324.6 kb) in the water flea *Daphnia magna*. J. Exp. Zool. B: Mol. Dev. Evol. *330*, 76-82.
10. Luchetti, A., Forni, G., Martelossi, J., Savojardo, C., Martelli, P.L., Casadio, R., Skaist, A.M., Wheelan, S.J., and Mantovani, B. (2021). Comparative genomics of tadpole shrimps (Crustacea, Branchiopoda, Notostraca): Dynamic genome evolution against the backdrop of morphological stasis. Genomics *113*, 4163-4172.
11. Orr, D.T. (2017). Genomic architecture of *Triops cancriformis* sex chromosomes in response to sexual system transition. *(Thesis).* (University of Hull).
12. Kim, H.S., Kim, B.M., Lee, B.Y., Souissi, S., Park, H.G., and Lee, J.S. (2016). Identification of *Hox* genes and rearrangements within the single homeobox (*Hox*) cluster (192.8 kb) of the cyclopoid copepod (*Paracyclopina nana*). J. Exp. Zool. B: Mol. Dev. Evol. *326*, 105-109.
13. Gramates, L.S., Agapite, J., Attrill, H., Calvi, B.R., Crosby, M.A., Dos Santos, G., Goodman, J.L., Goutte-Gattat, D., Jenkins, V.K., and Kaufman, T. (2022). FlyBase: a guided tour of highlighted features. Genetics *220*, iyac035.
